# *WFhb1-1* plays an important role in resistance against Fusarium head blight in wheat

**DOI:** 10.1038/s41598-020-64777-9

**Published:** 2020-05-08

**Authors:** Bimal Paudel, Yongbin Zhuang, Aravind Galla, Subha Dahal, Yinjie Qiu, Anjun Ma, Tajbir Raihan, Yang Yen

**Affiliations:** 1 Department of Biology and Microbiology, South Dakota State University, Brookings, SD 57007 USA; 2 Present Address: College of Agronomy, Shandong Agricultural University, Taian, Shandong 271018 China; 3 Present Address: Department of Entomology, University of Arkansas, Fayetteville, AR 72701 USA; 4 Present Address: Department of Molecular Genetics, University of Toronto, Toronto, ON M5S 1A8 Canada; 5 Present Address: Department of Horticultural Science, University of Minnesota, St. Paul, MN 55108 USA; 6 Present Address: Department of Biomedical Informatics, College of Medicine, The Ohio State University, Columbus, OH 43210 USA

**Keywords:** Gene expression profiling, Biotic

## Abstract

Fusarium head blight (FHB) is a severe disease of wheat (*Triticum aestivum* L.). *Qfhb1* is the most important quantitative trait locus (QTL) for FHB resistance. We previously identified wheat gene *WFhb1-1* (aka *WFhb1-c1*) as a candidate for FHB resistance gene. Here we report that *WFhb1-1* has been cloned. The gene (GenBank # KU304333.1) consists of a single exon, encoding a putative membrane protein of 127 amino acids. WFhb1-1 protein produced in *Pichia pastoris* inhibits growth of both *F. graminearum* and *P. pastoris* in culture. Western Blotting with anti- WFhb1-1 antibody revealed a significant decrease (*p* < 0.01) in WFhb1-1 accumulation, 12 hours post *Fusarium* inoculation in non-*Qfhb1*-carrier wheat but not in *Qfhb1*-carrier wheat. Overexpressing *WFhb1-1* in non-*Qfhb1*-carrier wheat led to a significant decrease (*p* < 0.01) in *Fusarium*-damaged rachis rate, *Fusarium*-diseased kernel rate and DON content in harvested kernels, while silencing *WFhb1-1* in *Qfhb1*-carrier wheat resulted in a significant increase (*p* < 0.01) in FHB severity. Therefore, *WFhb1-1* is an important FHB resistance gene with a potential antifungal function and probably a key functional component of *Qfhb1* in wheat. A model regarding how *WFhb1-1* functions in FHB resistance/susceptibility is hypothesized and discussed.

## Introduction

Fusarium head blight (FHB, also called scab or head scab), is a severe fungal disease of small grains such as bread wheat (*Triticum aestivum* L.), durum wheat (*T. durum* L.), oat (*Avena sativa*), and barley (*Hordeum vulgare* L.). FHB can be caused by several *Fusarium* species with *F. graminearum* as the primary pathogen in warm and humid regions worldwide including USA. Economic losses caused by FHB in wheat alone have been over billions of US dollar since 1990^1,2^. Reduced yields, shriveled grains, mycotoxin contamination, and reduction in seed quality are major factors that are related to the losses due to this disease^3–6^. The mycotoxins produced by the pathogen remain in processed foods causing health hazards in humans and animals^7^. Deoxynivalenol (DON) is the primary mycotoxin produced by *F. graminearum* in infected grains^3^.

Utilization of host resistance to develop resistant cultivars is the most promising approach to control FHB. Two major types of FHB resistance are widely accepted: resistance to the initial infection (Type I), and resistance to the spread of infection in the spike (Type II)^8^. Type I resistance is common in barley but rare in wheat, which is most likely contributed by spike morphology^9^ and by activation of systemic innate immune responses^10^. In contrast, Type II resistance is attributed by different resistant genes, and has been more extensively studied and utilized. FHB resistance in wheat is a quantitative trait. Numerous genetic studies on various resistance sources have shown that Type II resistance in each resistant wheat cultivar is most likely controlled by two to three major genes and a few minor genes^11,12^. Molecular mapping of quantitative trait loci (QTLs) for Type II resistance has been extensively reported. Overall, about 100 QTLs associated with FHB resistance are mapped in all wheat chromosomes but 7D^13^. Effectiveness of these QTLs is strongly influenced by genetic background and environments. Efforts to identify candidate genes of some key QTLs have also been made^14–21^, which has led to a better understanding of the pathogenesis and the resistance mechanisms.

The FHB-resistance QTL *Qfhb1* (formerly known as *Qfhs.ndsu-3BS* and sometimes simply called *Fhb1*) on chromosome arm 3BS was first identified from Chinese cultivar Sumai 3^22^. Since then, it has been well defined as the most effective and the most stable QTL across different genetic backgrounds and various environment^22–26^. *Qfhb1* usually account for 20~60% of the phenotypic variation in FHB resistance^13^. Therefore, *Qfhb1* has been the main resistance QTL deployed in wheat breeding to improve FHB resistance worldwide and the research focus for the resistance mechanism in wheat.

Fine mapping efforts have indicated that *Qfhb1* contains a complex chromosomal region dissimilar in the sequence between wheat accessions (see the review by Paudel and Yen^27^). In wheat cultivar Chinese Spring, this QTL was narrowed down to a 261-kb region of wheat chromosome arm 3BS^14,28–30^. Seven potential genes in this QTL region have been recognized, cloned and evaluated, but none of them was found to be an FHB resistance gene^14^. Later, 28 genes were recognized in a Sumai 3-derived, *Qfhb1*-containing 860-kb region, from which only a *GDSL* lipase gene showed a pathogen-dependent expression pattern and thus was thought to be qualified as a functional gene candidate for *Qfhb1* while a possibility of more than one gene causing the phenotypic difference was also suggested^16^. However, this *GDSL* was not among the 13 genes identified in the QTL interval of Sumai 3 by Pumphrey^31^ and Rawat *et al*.^17^. Rawat *et al*.^17^ instead claimed that a pore forming toxin like protein gene (*PFT*) is a functional gene of *Qfhb1*. Nevertheless, *Qfhb1* is able to detoxify DON^32,33^, but *PFT* cannot^17^. The unique existence of *GDSL* and *PFT* in *Qfhb1*-carrier wheat genotypes was a key reason for their identification as the genic component of *Qfhb1*. Su *et al*.^19^ and Li *et al*.^20^ independently did an extensive study by surveying hundreds of wheat accessions from worldwide collections and found that neither *PFT* nor *GDSL* is unique to *Qfhb1*-carrier wheat genotypes. Additionally, He *et al*.^34^ and Jia *et al*.^35^ also reported that *PFT* exists and functions in some susceptible wheat accessions they surveyed. These latest studies called into question the idea of *PFT* being an *Fhb1* candidate gene. In the most recent publications, Su *et al*.^19^ and Li *et al*.^20^ independently concluded that a mutation of a histidine-rich calcium-binding protein gene [named as *TaHRC* in Su *et al*.^19^ and *His* in Li *et al*.^20^] confers resistance against FHB. *TaHRC* is a susceptible gene, and a large deletion in the start codon region of its susceptible allele makes it silent, resulting in FHB resistance^19,36,37^. However, Li *et al*.^20^ claimed that the deletion in *TaHRC* had caused frameshift leading to expression of a new protein that confers resistance. Both Su *et al*.^19^ and Li *et al*.^20^ reported that the FHB resistance conferred by the deletions is genetically semi-dominant. Previously, we reported that a wheat gene with an unknown function, then temporarily named as *Wheat Fhb1 candidate 1* (*WFhb1-c1,* *WFhb1-1 or Fhb1-1*), could be a functional genic component of this QTL^15,23,38^. Therefore, the debate on *Qfhb1*’s genic component is going on.

Various functional mechanisms of *Qfhb1* have also been proposed, but none has been validated without argument. These proposed functions include detoxifying DON^32,33^, thickening secondary cell wall in rachises after pathogen infection to prevent the pathogen to spread^39^, inhibiting pectin methyl esterase to prevent the pathogen from penetrating the host cell wall^15^, mediating jasmonic acid (JA) and ethylene (ET) signaling pathways to elicit local and systemic resistance^16,38,40,41^, killing the infecting pathogen^17,42^ or simply reducing FHB susceptibility that leads to FHB development^37,43^. Possible simultaneous regulation of at least two different resistance mechanisms by multiple functional components of *Qfhb1* has also been suggested^16,29,40,43^. Nevertheless, *Qfhb1* has been well recognized to simultaneously reduce FHB severity in the spikes and DON content in the kernels.

As mentioned above, our previous studies using a combination of approaches including transcriptomics and physiological studies^38^, and QTL, eQTL, and physical mappings^15,23^ have revealed wheat gene *WFhb1-1* as a candidate for the functional genic component of *Qfhb1* in Sumai 3. Analyzing the expression pattern of this gene revealed its differential expression between FHB-resistant and FHB-susceptible wheat lines in response to the *F. graminearum* infection during the early stage of the pathogenesis^15^. This result implies that the pathogen can suppress the expression of this wheat gene to initiate FHB pathogenesis in FHB-susceptible wheat genotypes, but such suppression mechanism does not work in FHB-resistant wheat genotypes. Therefore, cloning of this gene, and elucidating its function are necessary to further our understanding about its role in FHB pathogenesis/resistance and about how it is regulated by the pathogen infection. Here we report the results of our efforts to clone this gene from Sumai 3, to elucidate its biological function and to functionally validate its role in FHB resistance using Sumai 3 and a pair of Sumai 3-derived *Qfhb1* near-isogenic lines 206-1-1-2 (carrying *Qfhb1*, called NIL-R hereafter) and 260-1-1-4 (not carrying *Qfhb1*, called NIL-S hereafter).

## Results

### Cloning the full-length cDNA and the genomic sequence of *WFhb1-1*

**Figure 1:**
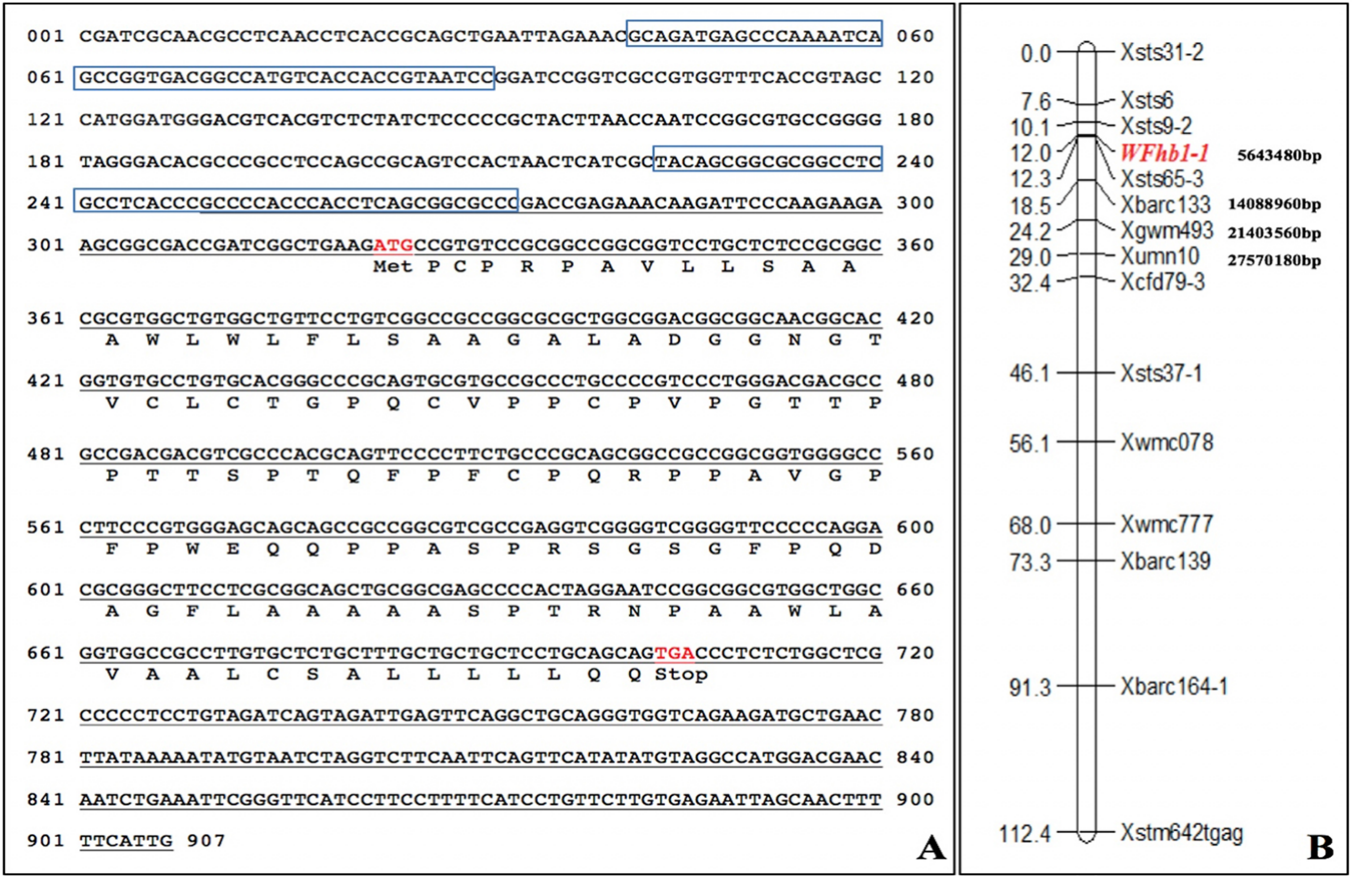
Sequences and map position of *WFhb1-1*. (**A**) Genomic sequence of *WFhb1-1* with the two predicted promoter regions in blue squares. The full-length cDNA is underlined and the predicted protein sequence is underneath. (**B)** *In silico* mapping of *WFhb1-1* to Chinese Spring chromosome 3B pseudomolecule with position in the sequence indicated.

The first step of this study was to clone the full-length cDNA of *WFhb1-1*, the candidate gene for a functional genic component of *Qfhb1*which we identified in our previous study^15^. The 5′ and the 3′ ends of the cDNA were first cloned from the spike sample of Sumai 3 with 5′/3′ RACE technologies, respectively, and the full-length cDNA sequence was then formed by merging the two partial cDNA fragments together. The full-length cDNA was then confirmed by cloning the full sequence from total RNA of Sumai 3 spikes by RT-qPCR and sequencing. The full-length cDNA sequence (Fig. 1A) has been deposited in GenBank (GenBank # KU304333.1).

The corresponding genomic sequence was then cloned from Sumai 3 by PCR with gene-specific primers designed on the basis of the cDNA sequences and the corresponding up-stream and down-stream genomic sequences of chromosome arm 3BS pseudomolecule (GenBank #: HG670306.1) of wheat cultivar Chinese Spring (now part of wheat reference genomic sequence IWGSC RefSeq v1.0.). Comparing the full-length cDNA and the genomic coding sequence revealed only one exon in this gene (Fig. 1A). Analyzing the upstream genomic sequence revealed two potential promoters (Fig. 1A). Our *in silico* mapping has located the cloned gene between the 5641624 bp and the 5642007 bp in the 3BS pseudomolecule flanked by markers *Xsts9-2* and *Xsts65-3* (Fig. 1B).

The predicted protein of this cloned gene (GenBank # ANE31719.1) has 127 amino acid residues, which shows 98% identity (*E* = 1e-80) to an unnamed wheat protein (GenBank #: CDM801516.1) reported by Choulet *et al*.^44^. Analyzing the predicted protein with Phobius (http://phobius.sbc.su.se) predicted that it is probably a transmembrane protein with an undefined extracellular signaling domain (Supplementary Fig. S1). However, analyzing it with ScanProsite (https://prosite.expasy.org/scanprosite/) did not give a clear clue on its biological function since no obvious conservative protein domain was found in the predicted protein. Therefore, we need to reveal the biological function of this protein experimentally.

### Protein expression in *Pichia pastoris*

**Figure 2:**
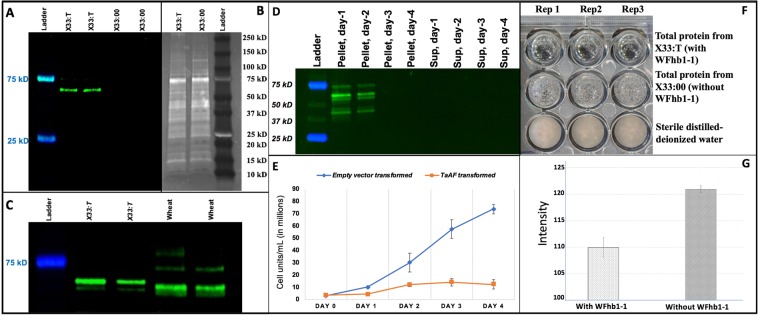
Photos and graphics showing the results of elucidating the WFhb1-1 produced by *WFhb1-1*-expression *Pichia pastoris* strain X33:T. (**A**) a photo of a Western Blot of total protein isolated from X33:T and the wild type *P. pastoris* X33:00 probed with anti- WFhb1-1 antibody PA-2; (**B**) a photo of a Sypro-Ruby stain-polyacrylamide gel of the total protein isolated from X33:T and X33:00; (**C**) a photo of a Western Blot of total protein isolated from X33:T and wheat spikes probed by PA-1. (**D**) A photo of Western Blot of total protein extracted from X33:T probed with anti- WFhb1-1 antibody PA-1 on day 1, day 2, day 3 and day4 after methanol was added into the culture to turn on *WFhb1-1* expression; Sup: supernatant. (**E**) A graphic showing the growth kinetics of X33:T and X33:00 post addition of methanol into the culture medium to turn on *WFhb1-1* expression. (**F**) A photo showing growth of *Fusarium graminearum* in 100 μL potato dextrose broth supplemented with 500 μg/mL total protein isolated from X33:T or X33:00 or sterile water. About 1000 conidia were used to initiate the culture in each well. The photo was taken two weeks after the culture started. (**G)** Comparison of intensity of fungal growth between WFhb1-1 protein-added and control protein-added wells.

To study the biological function of the cloned gene, its open reading frame (ORF) was cloned into and expressed in *P. pastoris* (Supplementary Fig. S2). Two antibodies PA-1 and PA-2 were designed and produced for specific detection of the putative protein encoded by the cloned gene (Fig. 1A). The yeast-produced WFhb1-1 was detected in the total protein extracted from the expressing yeast strain X33:T by Western blotting using PA-1 as the primary antibody (Fig. 2A). The yeast-produced WFhb1-1 stays intracellularly as it was detected in pellet samples but not in the supernatant of the cell extraction (Fig. 2D). The mass of the protein was estimated to be ~60 kDa (Fig. 2A–D), which is about 4 times larger than the ~13 kDa estimated from the predicted amino acid sequence. This observation indicate that this wheat protein probably forms a tetramer in the yeast total protein extraction. Alternatively, the yeast-produced WFhb1-1 may tightly bind to another yeast protein in the yeast cells.

We have noticed that the yeast production of WFhb1-1 could be detected only up to Day 2 of the induced expression (Fig. 2D), and growth of X33:T (indicated by its increased cell number per cubic unit of the culture) also occurred only during the first two days, while the control strain X33:00 continued to grow (Fig. 2E). These results suggest that the yeast-produced wheat protein may be toxic to *P. pastoris* itself so that it stops growing and ceases the production when WFhb1-1 concentration reaches the life-threatening threshold in two days.

### *In vitro* inhibition assay of *F. graminearum* growth with the yeast-produced WFhb1-1

Since the yeast-produced WFhb1-1 showed a potential anti-yeast activity in the expression system (Fig. 2E), we conducted experiments to see if it could also inhibit *F. graminearum* from growing in culture. Our data indicate that growth of *F. graminearum* in potato dextrose broth was indeed inhibited when 500 µg/mL or more of the total protein extracted from X33:T was added into the culture (Fig. 2F,G, Supplementary Fig. S3). The observed growth inhibition of *F. graminearum* by the total protein extracted from X33:00 yeast strain compared to the sterile water could be caused by precipitation reagent residues in the total protein sample. Nevertheless, the obvious difference in inhibiting *F. graminearum* growth by the X33:T total protein compared to that by the X33:00 total protein indicates that the yeast-produced WFhb1-1 seems to have a broad antifungal ability, which could reduce FHB development in wheat. Further research in this aspect with purified WFhb1-1 is needed to further explore its anti-fungal mechanism and its potential utility as a bio-fungicide for controlling fungal diseases.

### Evaluation of WFhb1-1 protein accumulation in wheat spikes

**Figure 3:**
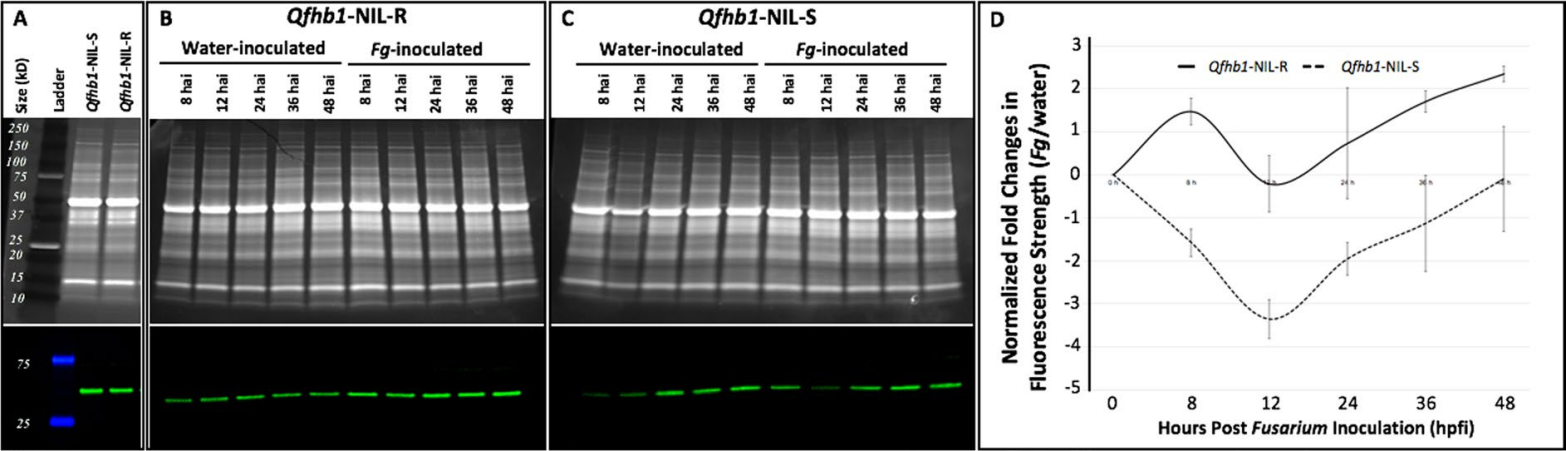
Photos and graphic showing the results of Western Blotting of total proteins isolated from wheat spikelets inoculated with *Fusarium graminearum* (*Fg*) or water at 0, 8, 12, 24, 36 and 48 hpfi (hours post *Fusarium* inoculation). In A, B and C, the upper panels are images of polyacrylamide gel stained with SYPRO Ruby, and the lower panels are photos of Western Blot probed with probed with anti- WFhb1-1 antibody PA-2. (**A**) Total protein isolated from the water-inoculated spikelets of *Qfhb1-*NIL-R and *Qfhb1*-NIL-S at 0 hpfi; (**B**) total protein isolated from the *Fg*- and the water-inoculated spikelets of *Qfhb1*-NIL-R; (**C**) total protein isolated from the *Fg*- and the water-inoculated spikelets of *Qfhb1*-NIL-S; (**D**) Normalized fold changes in fluorescence strength of WFhb1-1 revealed by Western blotting between the *Fg*-inoculated and the water-inoculated spikelets of each NIL at the six time points. The normalization was done by setting the value at 0 hpfi as the baseline for comparisons.

Western blotting assays using either anti-WFhb1-1 antibodies PA-1 (Fig. 2C) or PA-2 (Fig. 3) as primary antibody have detected WFhb1-1 accumulation in wheat spikes, confirming *WFhb1-1* as a protein coding wheat gene. Our previous study showed that *WFhb1-1* is suppressed by the pathogen infection in FHB-susceptible wheat genotypes but not in the FHB-resistant genotypes^15^. We would like to know how this transcription suppression impacts WFhb1-1 protein accumulation *in vivo*. Protein samples were collected from the *F. graminearum*-inoculated and the mock-inoculated spikelets of the NIL pair at 0, 8, 12, 24, 36 and 48 hpfi (hours post *Fusarium* infection) and subjected to Western blotting using anti-WFhb1-1 antibody PA-2 as the primary antibody (Fig. 3; Supplementary Figs. S4 and S5, Supplementary Table S1). We did not observe a significant difference at any time point between the FHB-inoculated and the mock inoculated samples of NIL-R (Fig. 3B,D, Supplementary Table S1). However, significant difference (*p* < 0.01) was observed at 12 hpfi between the FHB-inoculated and the mock-inoculated NIL-S spikes (Fig. 3C,D). These observations suggest that the pathogen infection reduces WFhb1-1 accumulation at the early stage of FHB pathogenesis but only in the FHB-susceptible wheat, similarly as we previously observed at the transcription level^15^. The estimated mass of the *in vivo*-produced WFhb1-1 is ~45 kDa (Figs. 2C & 3A), which is about three times larger than the ~13 kDa calculated from WFhb1-1 polypeptide sequence but smaller than the ~60 kDa estimated for the yeast-produced WFhb1-1 (Fig. 2C). The cause for the size difference between the yeast-produced, the wheat-produced and the calculated WFhb1-1 is unknown but could be due to formation of different multimers (i.e. trimer in wheat vs. tetramer in yeast). Binding to a protein of different size in the yeast vs. in wheat is an alternative explanation.

### *WFhb1-1* gene overexpression in wheat

**Figure 4:**
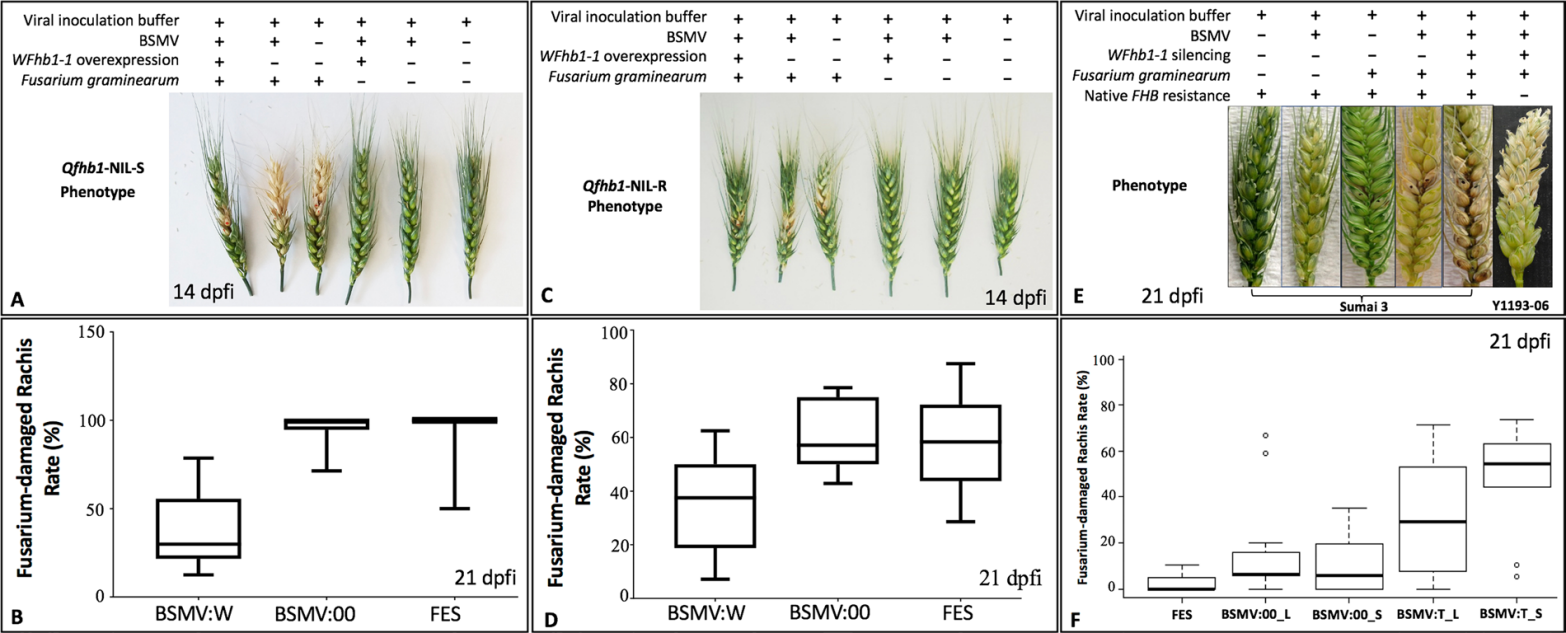
Photos and graphics showing the results of overexpression of *WFhb1-1* in near-isogenic lines *Qfhb1*-NIL-S (**A**,**B**) and *Qfhb1*-NIL-R (**C**,**D**) or silencing of *WFhb1-1* in FHB-resistant cultivar Sumai 3 (**E**,**F**) and FHB-susceptible landrace Y1193-06 (**E**) using a barley-stripe-mosaic virus (BSMV)-based system. Viral inoculation on spikes was applied. dpfi: days post *Fusarium* inoculation; FES: viral inoculation buffer FES; BSMV:00: wildtype BSMV; BSMV:W: *WFhb1-1*-overexpressing BSMV; BSMV:T: *WFhb1-1*-silencing BSMV; _L leaf inoculation of BSMV; _S: spike-inoculation of BSMV. The red or black dots on spikelets indicate the inoculated spikelets.

To understand the function of *WFhb1-1* in FHB resistance, we applied a barley stripe mosaic virus (BSMV)-based, virally-induced gene overexpression system to NIL-S and NIL*-*R to see how overexpression of *WFhb1-1* will impact FHB susceptibility/resistance in the NILs. To optimize this system in the NILs, the 3^rd^ leaves of 10-day old wheat seedlings were inoculated with either *WFhb1-1*-overexpressing BSMV strain BSMV:W or FES viral inoculation buffer. We first examined the existence of BSMV:W and the *WFhb1-1* expression by RT-qPCR in newly emerged leaves of the inoculated plants at 10 dpvi (days post viral inoculation). The BSMV was successfully detected in the BSMV:W-inoculated wheat plants but not in the FES-inoculated plants by RT-PCR, indicating that the infected BSMV:W was successfully assembled within the infected wheat leaves and were able to move to other parts of the plants. Since BSMV is known to replicate in the chloroplasts of the host cells, some degree of chlorosis can be seen in the BSMV-infected plant tissue (Fig. 4E) if the virus number is high. However, such chlorosis has minimum impact on the plant tissues and has never caused tissue necrosis under the experimental conditions.

**Figure 5:**
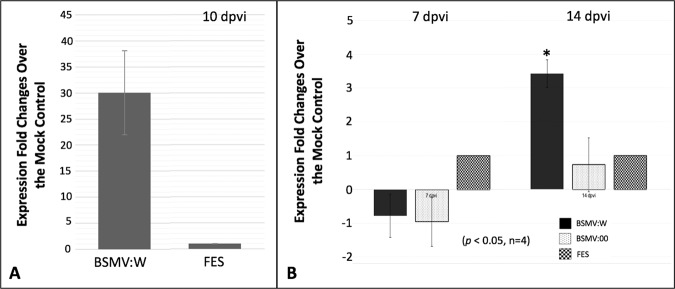
Results of RT-qPCR assays of *WFhb1-1* transcript abundance changes in leaves (**A**) or spikes (**B**) of the *Qfhb1*-NIL-S plants inoculated with the *WFhb1-1*-overexpressing strain (BSMV:W), wildtype BSMV (BSMV:00) in comparison with the viral inoculation buffer FES-inoculated mock control; dpvi: days post viral inoculation. Four biological repeats per treatment and three technical repeats per biological repeat were included in the analysis.

We detected up to ~30 fold increase *WFhb1-1* expression in BSMV:W-inoculated NIL-S plants compared to the FEF-inoculated NIL-S plants (Fig. 5A). This observation confirms that BSMV:W infection can induce overexpression of *WFhb1-1* in the infected NIL-S plants. We also tested direct BSMV:W inoculation in spikes of NIL-S plants when three fourth of a spike emerges from the flag leaf. In this experiment, wheat plants inoculated with BSMV:00 (the wildtype BSMV strain) or FES were used as the controls. We observed significant induction of the *WFhb1-1* overexpression in the spikes of the BSMV:W-inoculated plants compared to the BSMV:00- or the FES-inoculated plants (Fig. 5B). Results of this spike inoculation experiment suggest that significant overexpression could be detected at 14 dpvi.

**Figure 6:**
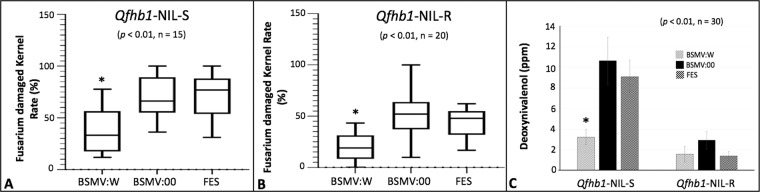
Graphics showing mean Fusarium-damaged kernel rate (%) (**A**,**B**) and deoxynivalenol content (ppm) (**C**) in the harvested kernels of *Qfhb1*-NIL-S and *Qfhb1*-NIL-R inoculated either with *WFhb1-1*-overexpressing BSMV:W, the wildtype BSMV:00 or the viral inoculation buffer FES. *Significantly different compared with others.

To understand if *WFhb1-1* indeed plays a role in FHB resistance in wheat, we visually observed FHB severity (Fig. 4A,C) on the inoculated spikes, and investigated *Fusarium* damaged rachides (FDR) rate (Fig. 4B,D, Supplementary Fig. S6), *Fusarium* damaged kernels (FDK) rate (Fig. 6A,B) and DON content in harvested kernels (Fig. 6C, and Supplementary Table S2) in the *WFhb1-1*-overexpressing wheat spikes under a high disease pressure (two spikelets were inoculated instead of usually one). For this purpose, NIL-S and NIL-R plants that were pre-inoculated with BSMV:W, BSMV:00 or FES were inoculated with *F. graminearum* conidia or water, respectively.

FDR data was collected from all the treated plants at 7, 14, 21, and 28 dpfi and analyzed (Supplementary Fig. S5). One-way ANOVA and T-test both showed that the FDR was significantly lower (*p* < 0.01) in the BSMV:W-inoculated spikes compared to the BSMV:00- or the FES-inoculated spikes in all the four time points for NIL-S and three of the four time points for NIL-R; FDR at 7 dpfi for NIL-R was not statistically significant (Supplementary Fig. S5). Similarly, FDK data was also collected for all the treated plants and it was also found significantly lower (*p* < 0.01) in the BSMV:W-inoculated spikes than in the BSMV:00- or the FES-inoculated spikes for both NILs (Fig. 6A,B). For NIL*-*S, the DON level was found significantly lower in the BSMV:W-inoculated spikes compared to the BSMV:00- or the FES-inoculated spikes, while no difference was observed among the treatments of NIL-R (Fig. 6C, Supplementary Table S2). It seems that overexpressing *WFhb1-1* in NIL-S reduces FHB to a level comparable to that observed in NIL-R (Figs. 5A,B & 6; Supplementary Fig. S6 and Table 2).

### *WFhb1-1* gene silencing in wheat

To further confirm *WFhb1-1*’s role in FHB resistance in wheat, the BSMV-system was also applied to knock *WFhb1-1* down in spikes of Sumai 3 and Y1193-06 with RNA interference. The reason for using these two wheat genotypes instead of the NILs in this experiment was that Sumai 3 has the strongest FHB resistance of all wheat genotypes that have been studied so far. It has not only *Qfhb1* but also other major FHB-resistance QTL, such as *Qfhb_6BL*^23^, *Qhfs.ifa*-*5A* (aka. *Fhb5*)^45–47^ and *Qfhb.mgb-2A*^21^, and it has been the most used FHB resistance source in wheat improvement worldwide. *WFhb1-1* could be a major FHB-resistance gene if knocking it down can significantly compromise FHB resistance in Sumai 3. Y1193-06 has the worst FHB susceptibility of all wheat genotypes we have evaluated so far, and, thus, is the best control in contrast to Sumai 3.

**Figure 7:**
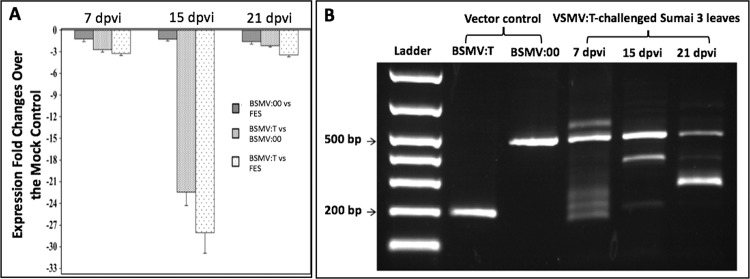
Results of RT-qPCR of *WFhb1-1* silencing (**A**) or PCR (**B**) assays of the stability of *WFhb1-1*-insert in BSMV:T viral vector in leaves of inoculated Sumai 3. Wildtype BSMV:00 and FES were used as the controls; dpvi: days post viral inoculation.

As we did in the *WFhb1-1* overexpression assay, we first tested if the knockdown works in Sumai 3. The leaves of Sumai 3 were inoculated with BSMV:T (the *WFhb1-1*-silencing BSMV strain), BSMV:00 or FES, and *WFhb1-1* expression was monitored at 7, 15 and 21 dpvi (Fig. 7A). Using leaves instead of spikelets here is totally for the convenience since we had already known that *WFhb1-1* is expressed in the whole plant and that the BSMV vectors will systematically spread to the entire plant body after inoculation.

Significant down expression of *WFhb1-1* was observed at 15 dpvi in the BSMV:T-inoculated plants compared to those inoculated with BSMV:00 or FES. *WFhb1-1* mRNA abundance in the BSMV:T-inoculated plants was 59~97% less than in the BSMV:00-inoculated plants. *WFhb1-1* mRNA was 17.1~37.5% less abundant in the BSMV:00-inoculated plants than in the FES-inoculated plants, which is statistically not significant (*p* > 0.05). As shown in Fig. 7B, the *WFhb1-1*-silencing insert in BSMV:T seems to be partially deleted from its viral carrier probably during the viral replication. This could explain why we observed a down-fall in *WFhb1-1* silencing at 21 dpvi (Fig. 7A). Code optimization seems to be needed to make the insert stable.

Then FHB inoculation was applied to the BSMV:T-inoculated and the control plants to test if silencing *WFhb1-1* in wheat spikes can increase FHB severity. In this experiment, BSMV:T was used for both leaf (this treatment was designated as BSMV:T_L) and spike (this treatment was designated as BSMV:T_S) inoculations in both Sumai 3 and Y1193-06. BSMV:00 and FES inoculations were conducted as the viral inoculation controls and sterile water was used as the mock *Fusarium* inoculant. FDR was monitored at 7, 15, 21 and 28 dpfi. Our data show that FHB resistance is compromised in the BSMV:T-treated Sumai 3 plants (Fig. 5E,F, Supplementary Figs. S6 to S8), which enabled *F. graminearum* to quickly spread to adjacent spikelets, whereas, FHB symptoms in the FES- and the BSMV:00-treated Sumai 3 plants were mainly limited to the inoculated spikelets (Fig. 5E; Supplementary Fig. S8). This successful breakdown of Type II resistance by *Fusarium* infection in BSMV:T-treated Sumai 3 plants could be phenotypically observed as early as 7 dpfi (Supplementary Fig. S8). In these plants, the disease seemed to spread mainly toward the base of the spikes, with only one or two upper rachis internodes being infected. Even at 28 dpfi, the upper spikelets remained clear of the fungal infection (Supplementary Fig. S8). As expected, no significant difference between treatments and control was observed in Y1193-06 (Fig. 6E; Supplementary Figs. S9 & S10). We noticed that the spikelets above the inoculation site on the BSMV:T + *Fusarium* inoculated spikes of Y1193-06 were dry to death (Fig. 5E) while this phenomenon was not observed on the BSMV:00+FHB inoculated Y1193-06 spikes. Therefore, knocking *WFhb1-1* down has enhanced FHB susceptibility in Y1193-06. Pearson’s product-moment correlation test using mean value of *WFhb1-1* abundance at 24 hpfi and FDR index data at 7 dpfi, 15 dpfi and 21dpfi collected from Sumai 3 indicated a strong negative association between the two (*p* = 0.00027 and *Cor* = −8.62). Our data indicate that viral inoculation on spike apparently is more effective in inducing RNAi than the leaf-inoculation (Supplementary Figs. S7 & S8).

### Evaluation of *WFhb1-1* expression in *TaHRC*-knockout Bobwhite mutant

CRISPR/Cas9-based gene editing of *TaHRC* in FHB-susceptible Bobwhite resulted in deletion mutations that knocked *TaHRC* out, gaining FHB resistance^28^. Expression of *WFhb1-1*, *PFT*, *GDSL* and *TaHRC* were compared between the *TaHRC*-knockout mutant created by Sue *et al*.^19^ and the wildtype of Bobwhite. Compared to the wildtype Bobwhite, *TaHRC* expression was found ~2.5 or ~3.58 folds lower, respectively using the gene-specific primers reported by Su *et al*.^19^ or Li *et al*.^20^, in the spikelets of the knockout plants 24 hours after FHB inoculation, while the expression of *WFhb1-1* was ~3 folds higher in the knockout mutant plants. Whereas, in both Bobwhite lines, expression of *PFT* was undetectable and *GDSL* expression level was too low to make any meaningful analysis. Therefore, both *PFT* and *GDSL* do not seem to have any role in the FHB resistance conferred by *Qfhb1*.

## Discussion

The goal of this study is to identify the functional component of the major FHB resistance QTL *Qfhb1* in wheat. We have taken a functional approach to reach this goal. We first conducted transcriptomics analysis between FHB-resistant and FHB-susceptible wheat lines and identified 637 genes that have significantly changed their expression abundance in wheat spikelets after *F. graminearum* infection and, therefore, are FHB-related genes^38^. Of these 637 genes, 406 genes which are associated with FHB resistance were analyzed by expression QTL (eQTL) mapping, and the significantly changed expression of three genes were found to be associated with *Qfhb1*^15^. Since these three genes can either physically locate in *Qfhb1* or physically locate at other places in the genome but are regulated by a gene in *Qfhb1*, we conducted a physical mapping using a series of nullisomic-tetrasomic lines of Chinese Spring to identify their physical locations in wheat genome and found that *WFhb1-1* is the only gene that is both physically located to wheat chromosome 3B where *Qfhb1* locates and functionally associated with *Qfhb1*^15^. In the present study, we have revealed the coding sequence of *WFhb1-1* (Fig. 1), elucidated its expression at protein level (Figs. 2 and 3), and transiently assayed its function in FHB resistance with overexpression and RNAi-induced silencing (Figs. 4 and 5).

Data obtained in the present study shows that *F. graminearum* infection causes a significant reduction of WFhb1-1 accumulation in NIL-S in the early hours of the disease development but not in NIL*-*R (Fig. 3). This observation is in line with our previous observation at the transcription level showing negative regulation of *WFhb1-1* by the infecting pathogen. Our data have also shown that overexpressing *WFhb1-1* leads to a significant reduction in FHB severity under a high disease pressure in both the NILs, and the FHB resistance level in the *WFhb1-1-*overexpressing NIL-S plants is comparable to the level usually observed in NIL-R (Figs. 4A–D and 6A,B; Supplementary Fig. S6). By contrast, silencing *WFhb1-1* leads to the significant compromise of FHB resistance in Sumai 3 and a noticeably increased FHB susceptibility in Y1193-06 (Fig. 4E,F; Supplementary Figs. S7 to S11). Overexpressing *WFhb1-1* also significantly reduces DON content in the kernels of NIL-S (Fig. 6C). Therefore, our findings show that *WFhb1-1* is a major FHB-resistance gene in wheat that can significantly reduce both FHB severity and DON accumulation even under high disease pressure. Wang *et al*.^48^ reported that *WFhb1-1* not only confers FHB resistance but also shows resistance against *Fusarium* root rot (FRR) by preventing the pathogen from spreading in the infected wheat plants. It seems that *WFhb1-1* works in the whole plant to protect it against *Fusarium*-caused diseases.

Our data show that *P*. *pastoris*-expressed WFhb1-1 protein can inhibit growth of both *P*. *pastoris* and *F. graminearum* in culture (Fig. 2D–G). These results suggest that WFhb1-1 is most likely an antifungal protein and probably functions to inhibit *F. graminearum* colonization *in planta*. Since our sequence analysis did not reveal any DON detoxification domain in WFhb1-1, the observed reduction in DON content in the kernels of the *WFhb1-1*-overexpressing NIL*-*S plants (Fig. 6C; Supplementary Table S2) is probably due to a reduced fungal population on the plants by WFhb1-1’s antifungal activity, not due to DON detoxification by WFhb1-1. It will be interesting to know if *WFhb1-1* has an even broader role in protecting wheat against more fungal diseases other than FHB and FRR.

**Figure 8:**
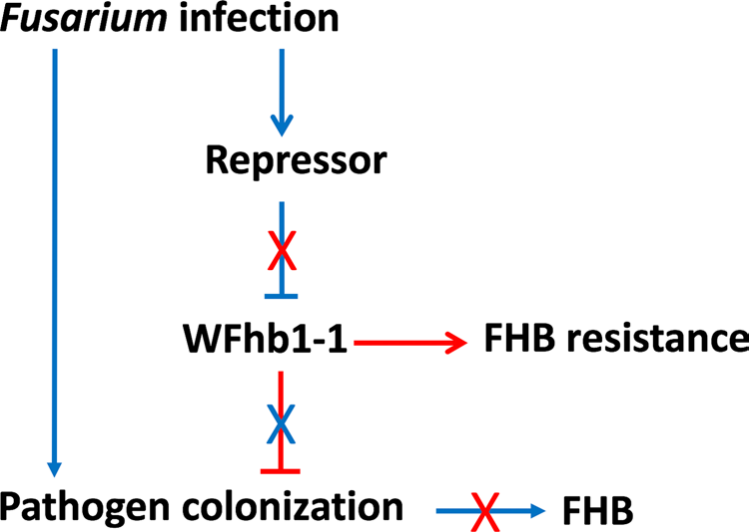
An illustration showing the hypothesis on how WFhb1-1 suppresses infecting pathogen leading to FHB resistance and how the infecting pathogen suppresses WFhb1-1 expression to gain colonization and thus develop FHB on the host plant. Arrowhead lines indicate promotion, T-headed lines denotes suppression, X means interruption, blue lines/letter lead to FHB development and red line/letter results in FHB resistance.

Our study has confirmed our previous conclusion that *F. graminearum* can suppress *WFhb1-1* expression in the early hours of FHB pathogenesis (Fig. 3; Supplementary Table S1). This suppression may be a key step in FHB resistance. In a previous study based on quantitative proteomic analysis of the same *Qfhb1* NILs, we found that FHB may result from a pathogen-promoted hypersensitive reaction (HSR) by the infected host cell, and *Qfhb1* largely functions to either alleviate HSR or to manipulate the host cells to not respond to the pathogen promotion^43^. The results from the present study suggests that the reduction of the pathogen population on the plant by WFhb1-1 might lead to significantly less infected host cells and thus significantly less HSR promotion in the host tissues by the pathogen. Therefore, as illustrated in Fig. 8, here we hypothesize a pathogen-host interaction model: *WFhb1-1* inhibits the growth of *F. graminearum* to prevent FHB development, the pathogen has developed an ability during the host-pathogen co-evolution to overcome *WFhb1-1*’s inhibition by suppressing its transcription during the initial stage of infection which leads to FHB development, and wheat then develops a currently unknown way to avoid the pathogen’s suppression resulting in resistance to FHB again. In this model, we hypothesize that the pathogen may either produce and then deliver a *WFhb1-1* suppressor into the host, or it may negatively regulate an indigenous host suppressor. In the first scenario, WFhb1-1 may work alone in contribution to FHB resistance. The second scenario actually fits to the multi-gene model of *Qfhb1*-confered FHB resistance proposed by Gao *et al*.^11^, Schweiger *et al*.^16^ and Rawat *et al*.^17^. The pathogen’s inability to suppress the *WFhb1-1* transcription from BSMV:W suggests that the suppression may target at the native *WFhb1-1* promotor or another regulation site but not *WFhb1-1* transcript itself. More research is needed to test this hypothesis and answer the question how FHB-resistant wheat genotypes escape this suppression.

Two recent publications have associated *TaHRC* with the *Qfhb1*-confered FHB resistance^19,20^. Although the resistance mechanisms reported in the two reports are controversial, both concluded that *TaHRC* is an FHB-susceptibility gene and a deletion in this gene has caused the loss of the FHB susceptibility. Su *et al*.^19^ reported that *TaHRC* is a nuclear protein with an unknown biological function. Therefore, it could be a regulator to other functional genic components of *Qfhb1*. In the recent study, we have found that knocking *TaHRC* out in FHB-susceptible wheat cultivar Bobwhite by CRISPR/Cas9 gene editing also causes upregulation of *WFhb1-1*. This result indicates that *TaHRC* might negatively regulate *WFhb1-1* expression. Therefore, it is highly probable that TaHRC is the *WFhb1-1*-suppressor in our model presented in Fig. 8, and in the cases reported by Su *et al*.^19^ or Li *et al*.^20^, a deletion in *TaHRC* causes the loss of its suppression of *WFhb1-1*, which results in a functional *WFhb1-1* during the pathogen infection and thus FHB resistance. Further researches should reveal how *TaHRC* regulates *WFhb1-1* and whether the pathogen indeed interacts with TaHRC to develop FHB.

As described by Zhuang *et al*.^15^, we previously identified *WFhb1-c1* as a candidate of the functional genic component of *Qfhb1* on the basis of the following evidences: (1) *WFhb1-1* was differentially expressed between NIL-S and NIL-R early in the pathogenesis^15,38^, which determines the FHB resistance or susceptibility in the NILs; (2) out of the 406 FHB-related genes investigated, *WFhb1-1* was the only one whose expression was significantly associated with *Qfhb1* by eQTL mapping^15^, which suggests that *WFhb1-1* either physically locates in the QTL or is functionally controlled by a gene in this QTL; and (3) nullisomic-tetrasomic analysis has physically mapped it on chromosome arm 3BS^15^. These arguments have been strengthened by the following findings from the present study: First, suppression of WFhb1-1 accumulation at the early stage of FHB pathogenesis was observed in NIL-S but not in NIL-R (Fig. 3); secondly, WFhb1-1 has been found to have antifungal ability (Fig. 2), thus, *WFhb1-1*’s role in FHB resistance seems to be an inhibitor to the pathogen growth *in planta*, which is well-aligned with *Qfhb1*’s function of conferring Type II resistance; and thirdly, FHB-susceptible NIL-S plants can be made as resistant as NIL-R plants are by simply overexpressing *WFhb1-1* in them, while silencing *WFhb1-1* in Sumai 3 resulted in complete compromise of FHB resistances (Fig. 4).

However, the present study still does not give a conclusive answer to the question whether *WFhb1-1* is indeed physically located in the *Qfhb1* interval or not. This is because *WFhb1-1* has been *in silico* mapped to a place between markers *Xsts9-2* and *Xsts65-3* in the 3BS pseudomolecule (Fig. 1B), which is found to be within the *Qfhb1* interval defined by some^15,23,29^ but outside the interval defined by others^16,17,31^. Particularly, *WFhb1-1* is not among the genes in the QTL interval reported by Schweiger *et al*.^16^ or by Rawat *et al*.^17^. This controversy could be at least partially due to sequence diversity in the *Qfhb1*-containing region among wheat cultivars. In fact, Schweiger *et al*.^16^ reported a high dissimilarity between Sumai 3 and Chinese Spring in the core region of *Qfhb1* that hosts the FHB marker UMN10 and the three published *Qfhb1* candidate genes: *GDSL*, *PFT*, and *HRC*. Furthermore, several landmark markers of this QTL have inconsistent positions on 3BS among the wheat accessions used in different mapping studies, indicating occurrence of inversions among these wheat accessions. For example, marker *Xbarc147* is moved from the proximal side of marker *Xgwm533* in the Sumai 3/Stoa map by Liu *et al*.^49^ to the distal side of *Xgwm533* in the Wangshuibai/Wheaton map by Yu *et al*.^50^ and the CS-SM3-3B/Annong8455 map by Zhou *et al*.^30^; *Xgwm493* is at the distal side of *Xumn10* in the Chinese Spring 3B pseudomolecule (Fig. 1B) but at its proximal side in the Sumai 3/Stoa map by Liu *et al*.^49^. Also, marker *Xbarc147* is at the distal side of *WFhb1-1* in our map^15^ and the *Qfhb1* interval in the Sumai 3/Stoa map by Liu *et al*.^49^ but in the middle of *Qfhb1* in the Wangshuibai/Wheaton map by Yu *et al*.^50^. Furthermore, *PFT*, the proposed candidate gene of *Qfhb1* by Rawat *et al*.^17^, was in fact mapped outside the QTL in FHB-resistant Wangshuibai and Sumai 3 according to Li *et al*.^20^. Therefore, it is possible that *WFhb1-1* and its regulator gene are both in the *Qfhb1* interval in some wheat lines but, in others, only the regulator is in the QTL with *WFhb1-1* itself flanking outside. Nevertheless, our data indicate that, being a genic component of *Qfhb1* or not, *WFhb1-1* is likely a key contributor to FHB resistance conferred by *Qfhb1* in wheat.

In summary, findings from this study demonstrate that *WFhb1-1* is likely a key FHB-resistance gene in wheat with potential antifungal function, and it most likely is a functional component of *Qfhb1* because *Qfhb1* cannot confer FHB resistance without *WFhb1-1*. Our data show that wheat needs a normal *WFhb1-1* expression at the initial stage of FHB pathogenesis to develop FHB resistance, while the pathogen needs to suppress *WFhb1-1* expression to colonize and thus cause FHB in wheat. Understanding how the pathogen suppresses *WFhb1-1* transcription will greatly help to our understanding of FHB pathogenesis and thus the control of FHB epidemics.

## Materials and Methods

The formulas of the buffers and media used in this study are listed in Supplementary Document S3. All the sequences of the adaptors and PCR primers used in this study are listed in Table 1.

**Table 1:** Sequences of adaptors and PCR primers used in this study.

Name	Sequence
*WFhb1-1* silencing insert Forward	TGTGCTCTGCTTTCCTGCTG
*WFhb1-1* silencing insert Reverse	CCCAGCATACAGTTGAAACG
*WFhb1-1* expression Forward	CTGAGCGGCTGCTGTGCTGA
*WFhb1-1* expression Reverse	ATATCAGATCTGGCAGTGCCCCA
BSMV:T stability Forward	TGATGATTCTTCTTCCGTTGC
BSMV:T stability Reverse	GTTTCCAATTCAGGCATCGT
pTMH Forward	TGCTACATCCATACTCCATCCTTCCC
pTMH Reverse	AGCTGACATCGACACCAACGATCT
UBC Forward	TCCCCTTACTCTGGCGGTGTC
UBC Reverse	TTGGGGTGGTAGATGCGTGTAGT
*WFhb1-1*-ORF-Forward	CACGCAGTTCCCCTTCTG
*WFhb1-1*-ORF-Reverse	GAGCAGCAGCAAAGCAGAG
5′AOX	GACTGGTTCCAATTGACAAGC
3′AOX	GCAAATGGCATTCTGACATCC
Gamma-1-Forward	CGCAATACGTAAGTCCGTAGC
Gamma-1-Reverse	GATGGGCACCATCAGATTT
Wheat β-actin Forward	AAATCTGGCATCACACTTTCTAC
Wheat β-actin Reverse	GTCTCAAACATAATCTGGGTCATC
*HRC-qPCR* Forward	CAGCAGAGTTCACACGATGA
*HRC-qPCR* Reverse	GGTGAGCCAGACAAGATGAA
*His* Forward	CAAGTACAGGCTTCAGAATCCA
*His* Reverse	GCAACTCGTGTAAGTTGTTAAAA
*PFT* Forward	GGATCTGGGCTGATTCAACT
*PFT* Reverse	TTTCGCAGAGCAATGAAGTC
*GDSL* Forward	TCAACAGGAGCCAGTTTGTC
*GDSL* Reverse	GATGTCCAAGGTGTAAAGCG

### Plant materials

Bread wheat cultivar Sumai 3 (FHB-resistant), landrace Y1193-06 (FHB-susceptible), a pair of *Qfhb1* NILs 206-1-1-2 (NIL-R) and 260-1-1-4 (NIL-S), Bobwhite (FHB-susceptible) and its CRISPR/Cas9-edited *TaHRC*-knockout mutant were used in this study. The NILs were developed and kindly provided by Dr. James Anderson’s lab at University of Minnesota. The *TaHRC* knockout Bobwhite mutant was kindly provided by Dr. Guihua Bai of USDA-ARS/Kansas State University. For each experiment, at least 10 plants per line per repeat per treatment were grown in pots filled with Miracle-Growth Potting Mix in a greenhouse or a growth chamber under a 16/8 h light/dark period, and 25/16 °C day/night temperature, supplied with cool, white fluorescent lamps.

### Rapid Amplification of cDNA ends (RACE) and Gene Cloning

RACE experiments were conducted using total RNA isolated from Sumai 3 and the 5’/3’ RACE Kit, 2^nd^ Generation (Roche Life Science). Gene-specific primers were designed according to the EST sequence (GenBank #: CA640991) that probe TaAffx.111425.2.S1_at on the Affymetrix Wheat Genome GeneChip was based on. The 5′ and 3′ RACE products were cloned and sequenced separately. The full-length cDNA was assembled by merging the 5′- and the 3′-amplicons from the RACEs, and then confirmed by PCR cloning of the whole sequence from Sumai 3. The cloned sequence was also compared with the publicly available chromosome arm 3BS pseudomolecule reference sequence of wheat cultivar Chinese Spring (now part of wheat reference genomic sequence IWGSC RefSeq v1.0.).

Genomic sequences of the gene of interest were PCR-cloned from Sumai 3 genomic DNA with primers designed based on the 3BS pseudomolecule sequence. Again, the PCR products of interest were cloned, sequenced and validated by comparing them to the relevant sequences in the 3BS pseudomolecule. Putative promoter sequences were predicted using TSSP and RegSite PlantProm DB (http://www.softberry.com/berry.phtml?topic=promoter) and Neural Network Promoter Prediction (http://www.fruitfly.org/seq_tools/promoter.html). Open reading frames were predicted by ORFfinder at NCBI.

### Prediction of protein property

Protein properties were predicted with Phobius, a combined transmembrane topology and signal peptide predictor at http://phobius.binf.ku.dk using the normal prediction function^51^. Analysis of conserved protein domains was done using quick scan mode of ScanProsite (http://prosite.expasy.org).

### Protein expression in *Pichia pastoris* and *in vitro* inhibition assay of *F. graminearum*

#### Antibody design

Primary antibodies were raised in rabbit against the peptides P Q R P P A V G P F P W E and Q Q P P A S P R S G S G F P, respectively, which were selected from the putative protein sequence of WFhb1-1 (Fig. 1A) following predictive analysis of protein folding. The antibodies were produced at GenScript USA Inc. The primary antibody against the protein motif P Q R P P A V G P F P W E was named PA-1, and the primary antibody against the protein motif Q Q P P A S P R S G S G F P was named PA-2.

#### Construction of expression vector

*WFhb1-1*’s ORF was synthesized according to the cloned gene sequence with an *EcoR* I site added at the 5′ end and a *Xho* I site added at the 3′ end. The synthesized foreign gene expression insert was put into *pUC57* and cloned into *Escherichia coli* JM 109 by heat shock method. Briefly, 1 µg *pUC57_insert* plasmid was added to 50 µl *E*. *coli* cells. The mixture was incubated on ice for 20 min, heat-shocked at 42 °C for 50 s and immediately kept on ice for 2 min. Then, 950 µl SOC medium was added into mixture followed by ~1.5 h incubation at 37 °C with shaking at ~150 rpm. The culture (50 µl or 100 µl) was spread on LB agar plates (with ampicillin 100 µg per ml) in duplicate. The plates were incubated at 37 °C overnight and plasmid DNA was extracted from developed colonies for further use.

EasySelect^TM^ *Pichia* Expression Kit (ThermoFisher Cat # K174001) was used to express the protein of interest in *P. pastoris* yeast. First, *pPICZA* was used to make the yeast expression vector. Briefly, the *pUC57-foreign gene* plasmid was digested with *EcoR* I and *Xho* I enzymes to release the expression insert, which was then ligated between the pre-cut *EcoR* I and *Xho* I sites in *pPICZA* using the T4 DNA ligase in 2X rapid ligation buffer from Promega. After the desired orientation of the insert in *pPICZA-foreign gene* was confirmed, nearly 10 µg of *pPICZA- WFhb1-1* was linearized with *Sac* I. The linearization was confirmed by running the digested and undigested plasmid parallelly in 1% agarose gel. For preparing competent cells of *P. pastoris* strain X33, the yeast cells were cultured in 5 mL YPD (yeast extract peptone dextrose) medium in a 50-mL conical flask at 29 °C overnight. Fifty milliliters of fresh YPD medium in a 250-mL conical flask was inoculated with 0.5 mL of the overnight culture and was again incubated at 29 °C overnight. Next day, the culture was centrifuged at 1500 × g for 5 min at 4 °C to harvest the cells. The supernatant was discarded, and the cells were resuspended in 50 mL of ice-cold, sterile water. The cells were again centrifuged as above and resuspended in 25 mL of ice-cold, sterile water. The cells were centrifuged again with the same speed and dissolved in 2 mL of ice-cold, sterile 1 M sorbitol. The cells were centrifuged one more time as above and dissolved in 0.5 mL of ice-cold, sterile 1 M sorbitol. Now the *P. pastoris* cells are ready for transformation by electroporation.

For transformation by electroporation, about 10 µg of the *Sac* I-linearized *pPICZA* expression vector was mixed with 80 μL of the *P. pastoris* competent cells and the mixture was transferred into an ice-cold 2-mm electroporation cuvette. The cuvette with the mixture was incubated on ice for 5 minutes. The cuvette was then put in BioRad GenePulsar X cell and pulsed once with pre-set protocol for *P. pastoris* with the parameters as follows: 2000 V, 25 µF, 200 Ώ and 2 mm. Immediately after the pulsing, 1 mL of ice-cold, sterile 1 M sorbitol was added in the cuvette, and the cuvette content was transferred into a sterile 15 ml tube. The tube was incubated at 29 °C without shaking for 1.5 hours. Then, the incubated culture in the tube was spread on YPD agar plates containing 100 µg/mL zeocin. Different volumes (10, 20, 50, 100, and 200 µL) of the culture were spread on separate YPD plates with zeocin. The plates were then incubated at 29 °C until the colonies formed (about a week). Then, 10 colonies were picked and the X33:T expression strain was further confirmed by its Mut+ phenotype following the protocol from Invitrogen (https://tools.thermofisher.com/content/sfs/manuals/easyselect_man.pdf). X33:T was also confirmed by PCR using *WFhb1-1*-ORF forward & reverse, 5′AOX and 3′AOX primers. X33 was also transformed with *pPICZA-wt* (the wildtype *pPICZA* plasmid without the expression insert) to form the background control strain X33:00, following the same protocol as explained above.

#### Protein expression in the *P. pastoris* expression system

For the protein expression experiments, a single colony of X33:T or X33:00 was cultured in 25 mL of MGY (minimal glycerol yeast) medium in a 250-mL conical flask and incubated at 29 °C for 16-18 h with shaking (250 rpm). To induce foreign protein expression in *P. pastoris*, cells were harvested by centrifuging at 3000 × g for 5 min at RT and the supernatant was discarded. The cell pellet was then resuspended in 25 mL of MM (minimal methanol) medium in a 250-mL conical flask, covered with two-layer sterile cheesecloth, and continued to incubate at 29 °C in the shaker incubator (250 rpm). Methanol was added to a final concentration of 0.5% every 24 hours to maintain the expression induction. At each time points (Day0, Day1, Day2, Day3, and Day4), 1 mL of the expression culture was collected into a 1.5 mL microcentrifuge tube. This sample is used to analyze the growth kinetics by counting the cells and to extract the total protein.

#### *Pichia pastoris* growth kinetics

*P. pastoris* growth kinetics were studied by counting the number of cells per milliliter in the expression culture in Day0, Day1, Day2, Day3 and Day4 starting from the time when methanol was first added into the MM medium (Day0). This was done by first diluting 10 µL of the culture from each time point with 990 µL of sterile water to make a 100-fold dilution. The diluted culture samples were then microscopically observed on a hemocytometer to count the cells in the four squares with 0.1 mm^3^ per square. The cell numbers in the four squares were averaged and multiplied with the dilution factor to get the final count as “cells /mL”. The budding cells attached together were counted as a single cell. This experiment was repeated for three times.

#### Protein extraction and quantification

Total proteins were extracted from both X33:T and X33:00. One milliliter of the expression culture sample was collected into a 1.5 mL microcentrifuge tube at each time point and centrifuged at maximum speed in a tabletop centrifuge for 2 minutes at room temperature. The supernatant was transferred into a new 1.5 mL tube. Both the supernatant and the pellet were snap-frozen in liquid nitrogen and stored at −80 °C until further processed. Total protein concentration of this supernatant was measured at 562 nm absorbance using Pierce BCA Protein Assay Kit from Thermo-Scientific, and final concentration of 1 µg/µL was maintained for all the samples by adding adequate MM medium.

For the extraction of total protein from pellet sample, each pellet was resuspended in100 µL breaking buffer. Equal volume of acid-washed glass beads (size 0.5 mm) was then added into the pellet suspension and vortexed for 30 s. The mixture was incubated on ice for 30 s and then vortexed before another 30 s incubation. This cycle was repeated for eight times. After the final vortexing, the mixture was centrifuged at maximum speed for 10 min at 4 °C. The clear supernatant was then transferred to a fresh 1.5 mL microcentrifuge tube. Total protein concentration was measured at 562 nm absorbance using Pierce BCA Protein Assay Kit from Thermo-Scientific, and 1 µg/µL final concentration was achieved by adding breaking buffer.

For extraction of total protein from wheat spikelets, each sample harvested at a desired time point were ground in 1.5 mL tubes with plastic pestle in liquid nitrogen until the fine powder was obtained. After grinding the sample, 200 µL of breaking buffer was added in each tube. Vortexing was done with intermittent incubation of tubes on ice (vortexing for 30 s followed by incubation on ice for 30 s for total 8 cycles). Then, the tubes were centrifuged at maximum speed at 4 °C for 10 min, and the clear supernatant was transferred into new micro-centrifuge tubes. Protein concentration was measured with 280 nm absorbance, and the concentration was also confirmed using Pierce^TM^ BCA Protein Assay Kit (ThermoFisher Cat # 23250). Additional breaking buffer, if needed, was added to make the final concentration of each protein sample 1 µg/µL. These extracted protein samples were stored at −80 °C until further used.

#### *In vitro* inhibition assay of *F. graminearum* with the yeast-produced protein

The total protein in breaking buffer in a 1.5-mL microcentrifuge tube was precipitated by adding four times (in volume) of cold acetone (stored at −20 °C) into the tube. The tube was then vortexed and incubated for 1 h at −20 °C. Then, the tube was centrifuged for 10 minutes at 15000 × g, and the supernatant was carefully discarded. The protein pellet was air-dried by leaving the tube lid open at RT for about 30 min. The dried pellet was then dissolved in adequative amount of sterile distilled water to make the total protein solutions of desired concentrations, which was measured by the standard BSA protein assay. Series of total protein concentrations (20, 50, 100, 500, 600, 700 µg/mL) in each volume were made and used in the inhibition assay. To produce a larger amount of total protein, the scale was increased by 50 folds.

To assay if the yeast-produced protein can inhibit *F. graminearum* growth, about 10,000 *F. graminearum* conidia were grown in 100 µL of potato dextrose broth (PDB) per well in a 48-well cell culture plate. Total protein extracted from either X33:T or X33:00 cultures in a concentration of 20, 50, 100, 500, 600 or 700 µg/mL was added into three parallel wells, and sterile water was used as blank control. Each treatment was repeated three times. Growth of *F. graminearum* in each well was visually assessed in two weeks after the treatment started, and high resolution pictures for plates were taken. The intensity of the fungal growth in each well was calculated and analyzed by using ImageJ software^52^. In brief, equal area of each wells was selected and the intensity of the selected area was calculated in ImageJ for each wells with fungal growth and the statistical analysis was done.

### Polyacrylamide gel electrophoresis and Western blotting

For SDS-PAGE, 25 µg of total protein was loaded in each well of BioRad pre-casted mini polyacrylamide gels. Precision plus protein standard (BioRad) was used to estimate the protein mass. The gel was run for about 1 h with 30 mA constant current in a Bio-Rad mini PROTEAN tetra cell, and then was either processed for Sypro-Ruby staining or for the Western blotting.

For Sypro-Ruby staining, the gel was first fixed in fixing solution (50% methanol and 10% acetic acid in distilled water) for 1 h. Then, the fixing solution was discarded, and the gel was soaked in 50 mL Sypro-Ruby protein gel stain solution for 2 h with gentle agitation and protection from light. Then the gel was transferred into a clean tray and washed with wash solution (10% methanol and 7% acetic acid in distilled water) with gentle shaking for 15 min. The gel is then visualized and recorded under UV.

For Western blotting, after the SDS-PAGE was run, the total protein on the gel was transferred onto a nitrocellulose membrane using iBlot from Invitrogen. Then, the membrane was incubated in buffer TBST with 5% dry milk for 1 h with gentle shaking for blocking. The membrane was then washed three times with buffer TBST having 0.5% dry milk (10 minutes each time with gentle shaking). Then the membrane was incubated in primary antibody solution diluted to 1:10,000 in TBST with 0.5% dry milk for 1 h at RT with gentle shaking. Then the membrane was washed for 3 times with TBST having 0.5% milk as described above. LICOR IRdye 800 CW goat anti-rabbit antibodies were used for secondary binding and visualization. Same dilution as primary antibodies (1:10,000) was used in TBST with 0.5% dry milk, and the membrane was incubated at RT for 1 h with gentle shaking. The membrane was then washed again 3 times as described above and visualized in LICOR Odyssey Fc under 800 nm absorbance with exposure time of 2 milliseconds.

Western Blots of total wheat protein were digitized for fluorescence signal strength with LICOR Odyssey Fc and analyzed. At least three bio-repeats per treatment and three technical repeats per bio-repeat were performed. For each NIL, the signal data were compared between the treatment and the mock control for each time point and the relative change were then normalized by taking the value at 0 hpfi (hours post *Fusarium* inoculation) off (Supplementary Table S1).

### Constructing overexpression and knockdown vectors

A barley stripe mosaic virus (BSMV)-based virus-induced gene overexpression (VOX)/silencing (VIGS) system was used to transiently overexpress or silence the gene of interest. The BSMV vectors used were kindly provided by Dr. Li Huang of Montana State University. The BSMV γ vector (γ PCR vector) was modified by Huang’s group with two *Xcm* I restriction sites inserted. We further modified it by adding an *EcoR* I site between the two *Xcm* I sites. To construct the overexpression vector BSMV:W, *WFhb1-1*’s ORF (384 bp) with a *EcoR* I site at each end was synthesized by GenScript. BSMV:W was then constructed by digesting the γ PCR vector and the synthesized ORF with *EcoR* I, and mixed then together to have the ORF inserted in the *EcoR* I site. The desired orientation of the ORF was confirmed with PCR using *WFhb1-1*-ORF forward/Gamma-1 reverse primers.

To produce RNAi silencing BSMV vector VIGS:T, TA cloning site was first produced from the γ PCR vector by digesting it with *XcmI*. A fragment of 289-bp complementary to the corresponding *WFhb1-1* coding sequence was amplified by PCR from Sumai 3 with *WFhb1-1* silencing primer set (Table 1) and ligated into the γ PCR vector. Both the VOX and VIGS vectors were confirmed by sequencing.

### *In vitro* transcription of viral RNA and plant inoculation

The three BSMV RNA chromosomes were reverse transcribed from the corresponding BSMV vectors following the protocol provided by Dr. Li Huang of Montana State University. RNA quality was assessed on 1% agarose gel. Virus inoculation was done by following the previously described inoculation procedures^53,54^. Briefly, a mixture of the three viral RNAs in viral inoculation buffer FES was manually rubbed into plant tissue.

Viral inoculation was carried out with either the VOX vector BSMV:W, the VIGS vector BSMV:T, the empty BSMV control vector BSMV:00 or the viral inoculation buffer FES alone on at least 10 plants per line per treatment. The inoculation was done either on a spike at shooting stage as soon as three fourths of the spike was emerged, on a 10-day old leaf, or on a flag leaf at the booting stage when the flag leaf was fully expanded.

### Fungal inoculum preparation, inoculation, sampling and disease evaluation

*F. graminearum* isolate Fg4, collected from Watertown, SD was used in this study to induce FHB. *F. graminearum* was cultured on potato-dextrose-agar (PDA) medium for a week, and then spores were collected for plant inoculation. Procedures used for wheat spike inoculation as previously described^34^. Briefly, *F. graminearum* spores were washed from PDA plates using sterile water and then filtered through four layers of sterile cheesecloth. The concentration of conidia was counted using a hemocytometer and adjusted with sterile water to about 100,000 conidia/mL. The spikelet was challenged with 10 μL of water-suspension of *F. graminearum* conidia or sterile water alone (as a mock control) at the stage when intensive yellow color of anthers was observed. For each treated spike, the two first-flowering spikelets were inoculated to introduce a disease pressure at the level that an FHB-resistant genotype will be maximumly diseased at 28 dpfi. The inoculated spikes were immediately covered with plastic zip-lock bags with a wet cotton ball inside for 72 h to maintain the needed humidity and temperature inside the bags (greenhouse effect) to facilitate disease establishment. The length between the BSMV and the *F. graminearum* inoculations was optimized for both leaf and spike inoculations by test inoculation of the two at various time intervals until the maximum effect of overexpression or knockdown of *WFhb1-1* was reached, usually at 4~7 dpvi when yellow pollens emerged out of the first pair of spikelets.

For disease evaluation, FDR was calculated as percentage of diseased rachides of all rachides per spike and FDK was calculated as percentage of diseased kernels of all harvested kernels per spike. FDR were averaged per time-point per treatment per experiment at 7, 14/15, 21 and 28 dpfi, respectively. DON content in the harvested kernels per spike was also measured by sending the harvested kernels to the DON testing lab at University of Minnesota and analyzed for each treatment in our lab.

### Quantitative Real-time PCR (RT-qPCR)

Total RNA was extracted from leaf, spike or spikelet samples with TRIZOL (Invitrogen) following the manufacturer’s instructions. RNA quality was tested using 0.8% or 1% agarose gel and quantified using a NanoDrop ND-1000 UV-Vis Spectrophotometer (Wilmington, DE). For each treatment, three to four biological replications were conducted. For reverse transcription, ~500 ng DNase I-treated total RNA was used for cDNA synthesis using GoScript™ Reverse Transcriptase system (Promega) with oligo(dT)_15_ primer. For VOX and VIGS experiments, if relevant, the presence of BSMV viral genome in the cDNA samples was confirmed before RT-qPCR was conducted. The RT-qPCR was conducted on a Smart Cycler II (Cepheid) or QuantStudio 6 Flex (Applied Biosystems). Briefly, 2X dilutions were made for reverse transcription products, and 1 μL diluted cDNA/20 μl reaction was carried out using SYBR green I master Mix with 2 min at 95 °C, 45 cycles of 20 s at 95 °C, 30 s at melting temperature, and 30 s at 72 °C, and then 5 min at 72 °C. Wheat β-actin gene was used as an internal control to normalize the *Ct* value. For each sample, three technical replications were conducted. Fold changes were calculated with the 2^−∆∆*Ct*^ method^55^.

### Statistical analyses

Tukey’s multiple comparisons of means in *R* software package was used to compare FDR and FDK values of each treatment at the 95% family-wise confidence level. We also conducted student *t-*test and one-way ANOVA analysis for these data. The association between transcript abundance of a gene of interest and FDR in the gene silencing experiment was measured using Pearson’s product-moment correlation analysis in *R* software package.

## Supplementary information

Supplementary information.
