# Supplementary material for: WFhb1-1 plays an important role in resistance against Fusarium head blight in wheat
Source: Sci Rep. 2020 May 8;10:7794. doi: 10.1038/s41598-020-64777-9 (PMC7210279; doi:10.1038/s41598-020-64777-9)
Supplement: Supplementary file 1 — Supplementary information. [file 41598_2020_64777_MOESM1_ESM.pdf]

WFhb1-1 plays an important role in resistance against Fusarium head blight in wheat

Bimal Paudel, Yongbin Zhuang, Aravind Galla, Subha Dahal, Yinjie Qiu, Anjun Ma, Tajbir Raihan, & Yang Yen

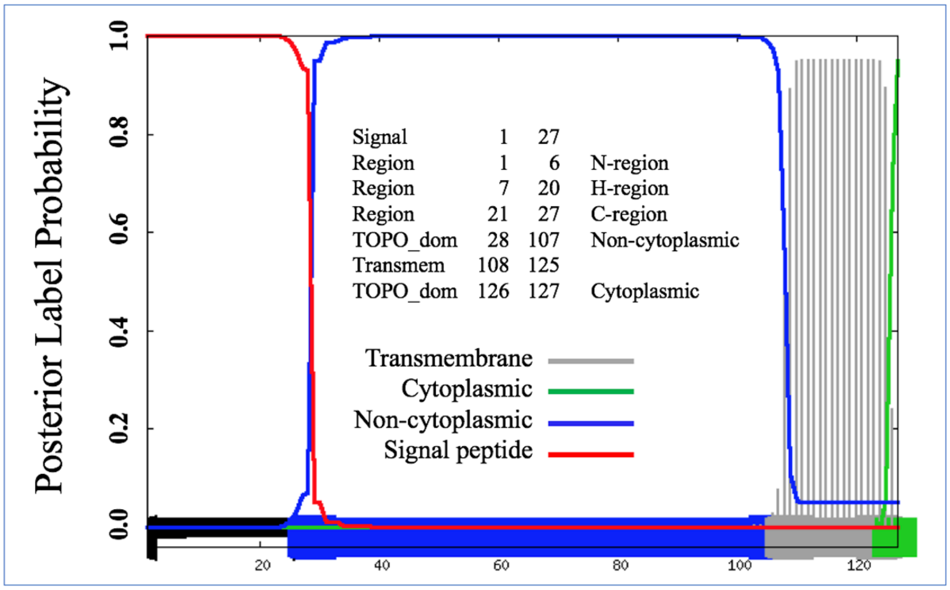

Supplementary Figure S1. A graphic illustrating the properties of WFhb1-1 protein predicted by Phobius.

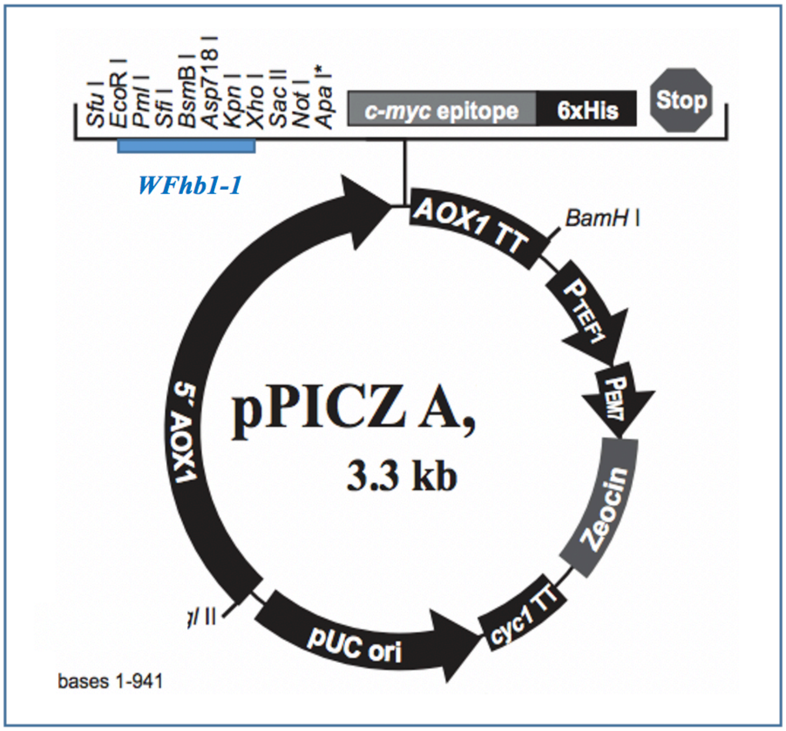

Supplementary Figure S2. An illustration of the *WFhb1-1* cloning position in *pPICZA* plasmid.

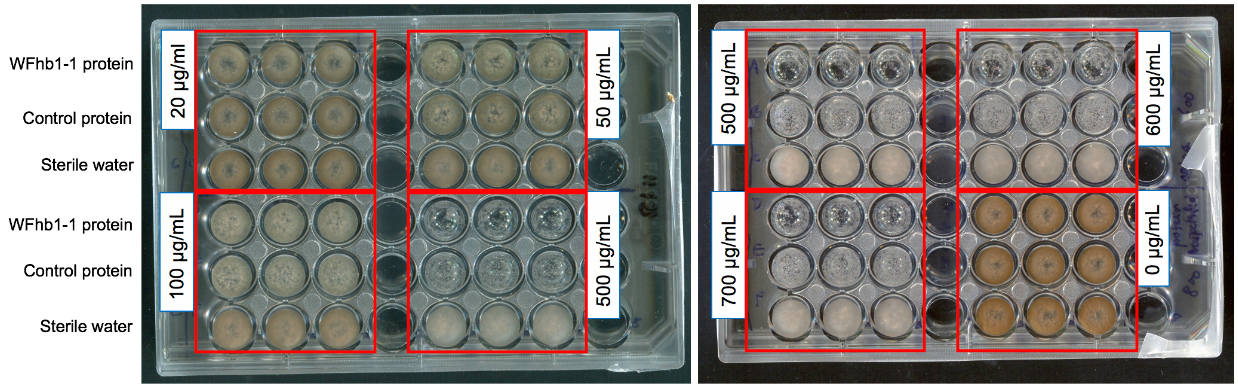

Supplementary Figure S3. Photos showing growth of 1000 conidia of *Fusarium graminearum* in 100 µL potato dextrose broth supplemented with, 0, 20, 50, 100, 500, 600 or 700 µg/mL total protein isolated from *WFhb1-1* -expression *Pichia pastoris* X33:T, wildtype X33:00 or sterile water. The photo was taken two weeks after the culture started.

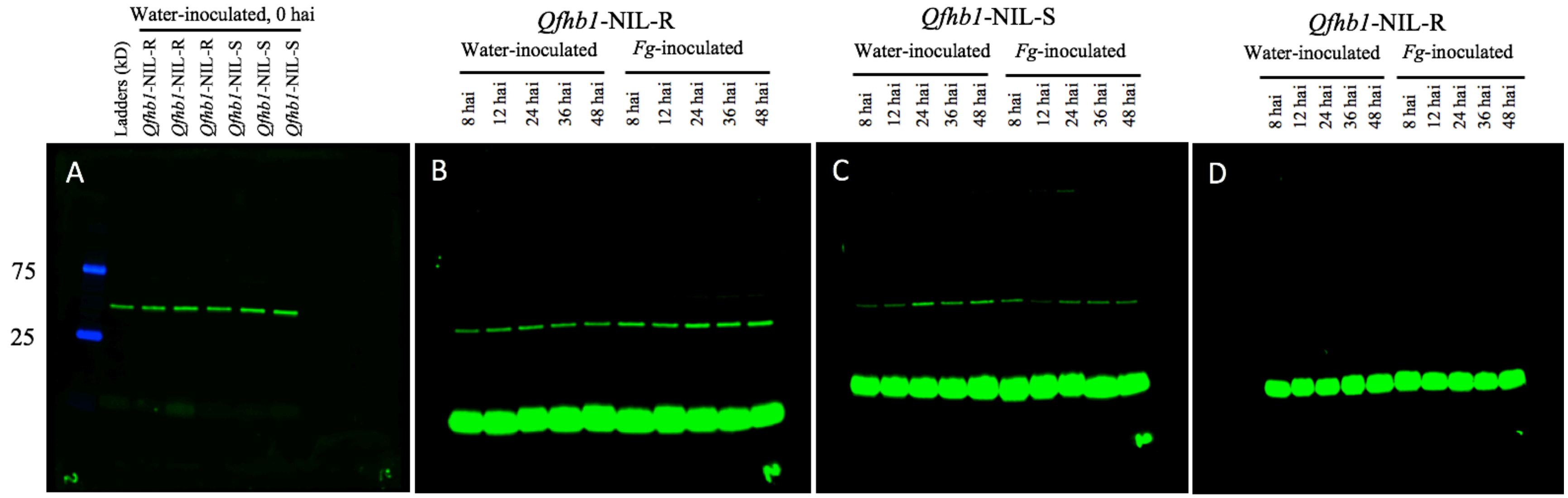

Supplementary Figures 4. The original photos used to prepare panels A, B and C in Figures 3, respectively. D: a gel loaded with the same samples as in B but was not hybridized with anti-WFhb1-1 antibody. *Fg*: *Fusarium graminearum*. hai: hours after inoculation.

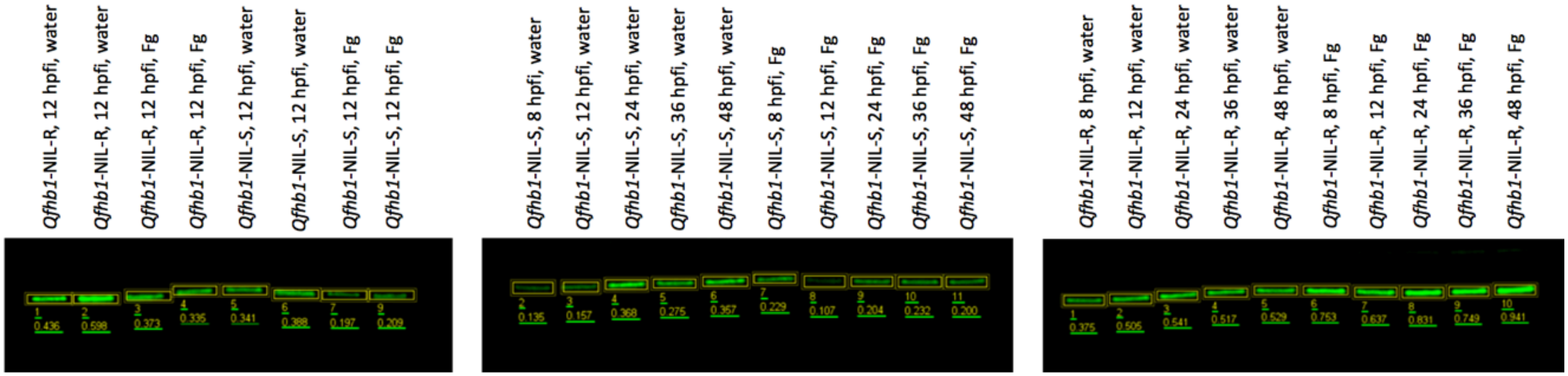

Supplementary Figure S5. Photos of representative images of digitizing fluorescence signal strength for each band on the Western Blots of WFhb1-1 protein probed with anti-WFhb1-1 antibody PA-2.

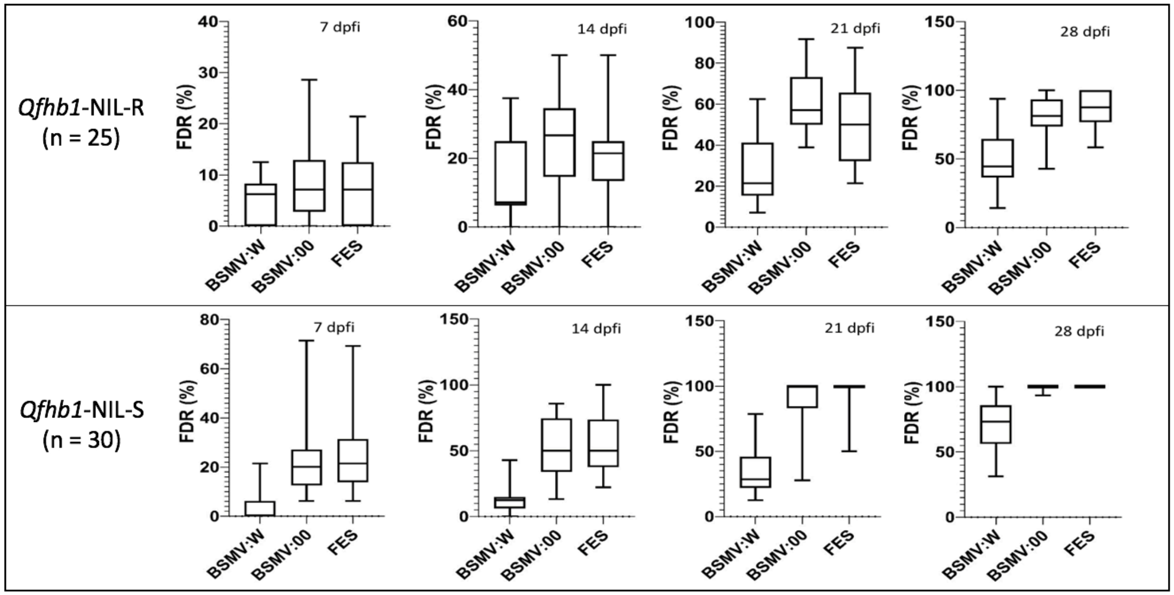

Supplementary Figure S6. Graphics showing mean *Fusarium* damaged rachis rate (FDR)) of *Qfhb1*-NIL-S and *Qfhb1*-NIL-R of the *WFhb1-1*-overexpression plants (BSMV:W) and controls (BSMV:00 and FES) in 7, 14, 21 and 28 days post FHB-inoculation (dpfi).

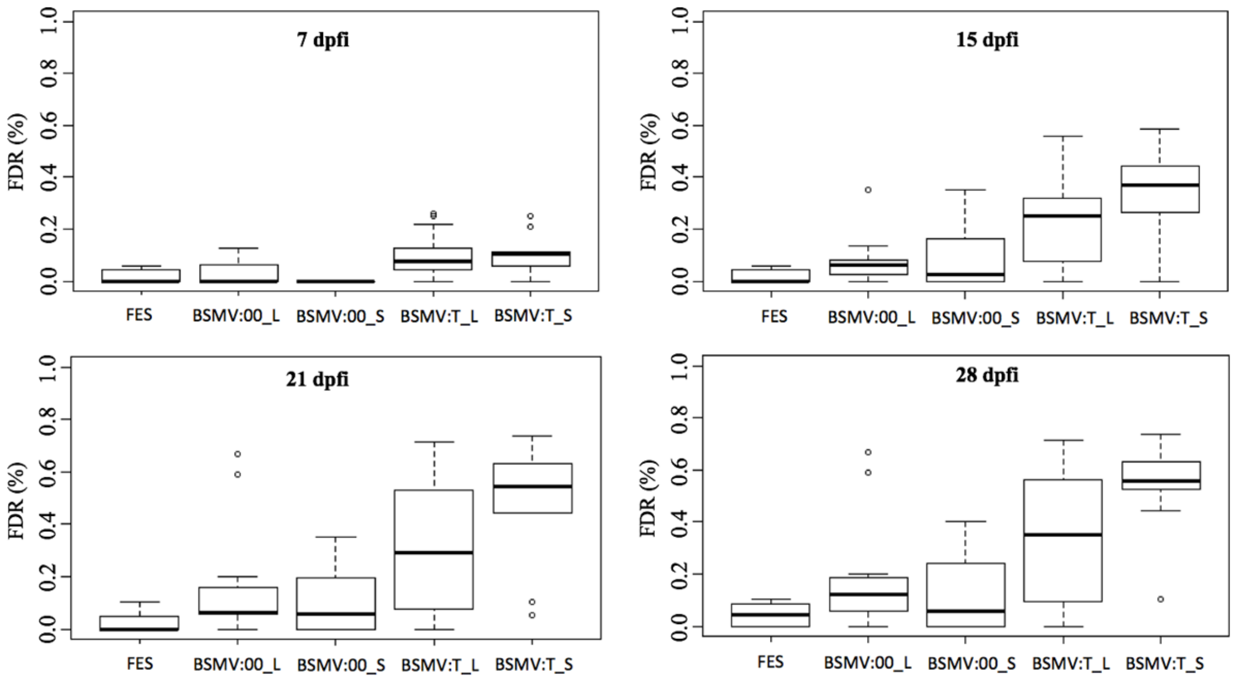

Supplementary Figure S7. Graphics showing mean FHB-damaged rachis rates (FDR) of Sumai 3 plants inoculated first with the inoculation buffer (FES), the empty vector control (BSMV:00) or *WFhb1-1*-silencing vector (BSMV:T) and then with *Fusarium graminearum* in 7, 15, 21 and 28 days post *Fusarium* inoculation (dpfi). _L: leaf viral inoculation; _S: spike viral inoculation.

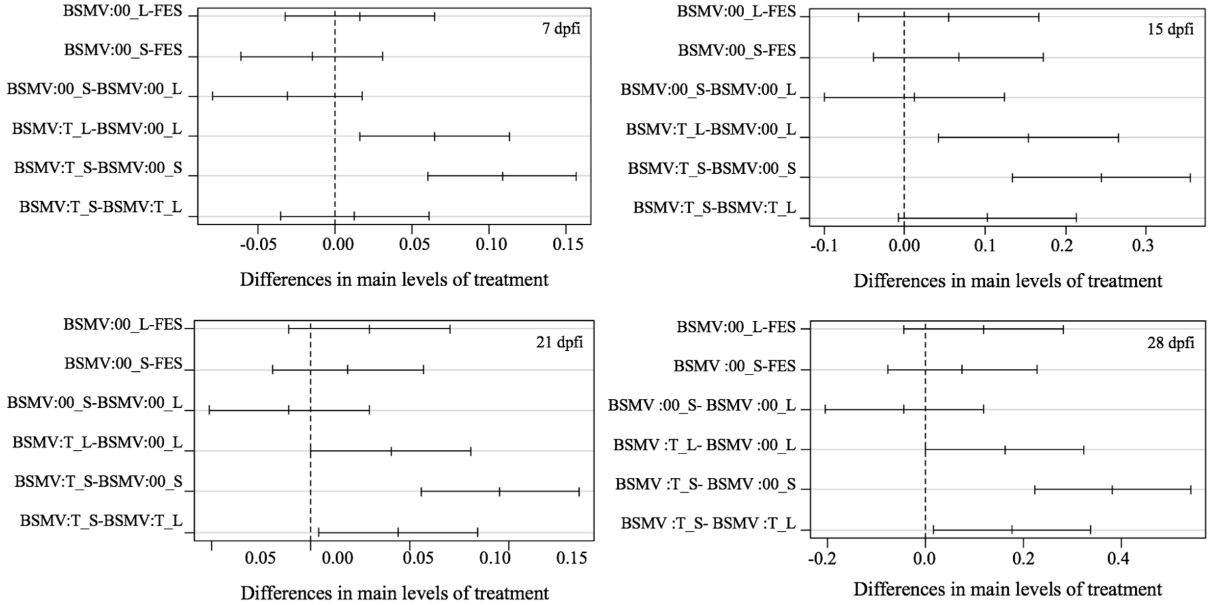

Supplementary Figure S8. Graphics showing the results of Turkey multiple comparisons of means between VIGS treatments at 95% family-wise confidence level for Sumai 3 in 7, 15, 21 and 28 days post *Fusarium* inoculation (dpfi).

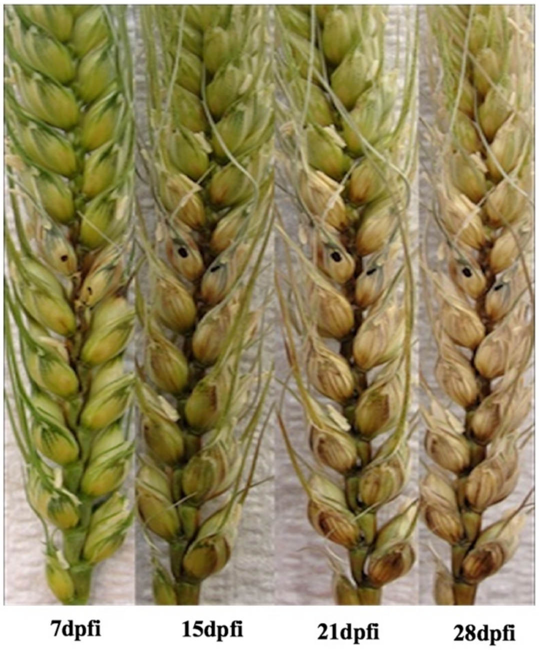

Supplementary Figure S9. Photos show typical phenotypes of Sumai 3 spikes inoculated first with *WFhb1-1*-silencing BSMV:T and 15 days later with *Fusarium graminearum*. dpfi: days post *Fusarium* inoculation.

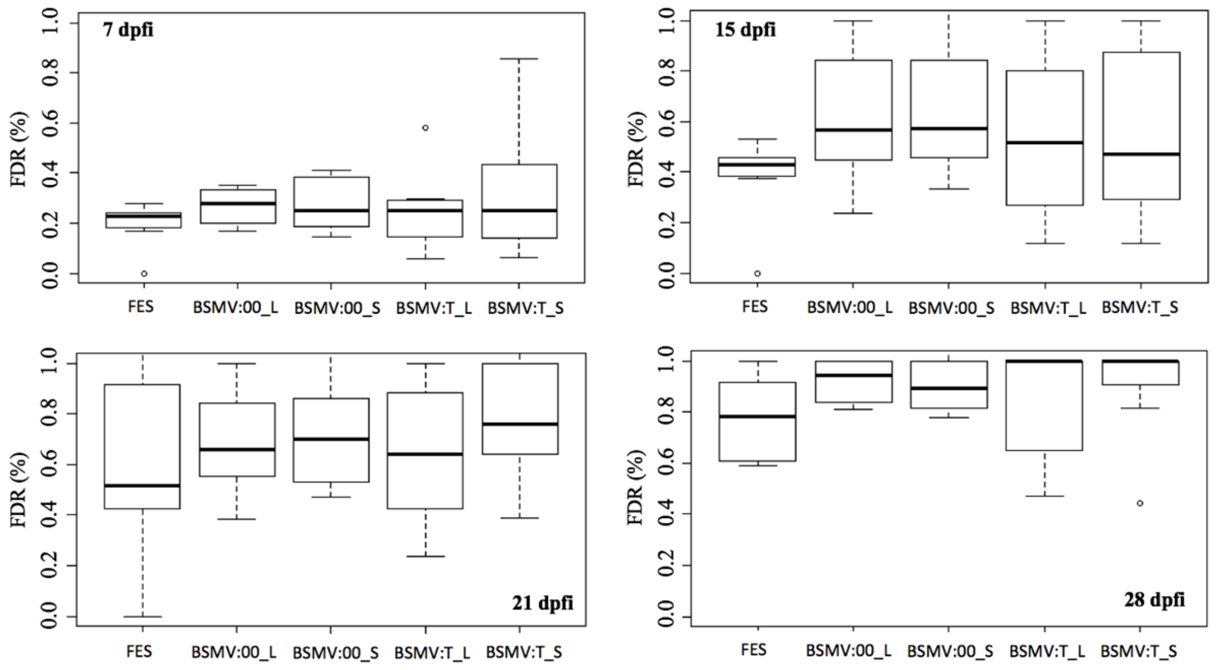

Supplementary Figure S10. Graphics showing mean FHB diseased rates (FDR) of Y1193-06 plants inoculated first with the inoculation buffer (FES), the empty vector control (BSMV:00) or *WFhb1-1*-silencing vector (BSMV:T) and then with *Fusarium graminearum* in 7, 15, 21 and 28 days post *Fusarium* inoculation (dpfi). _L: leaf viral inoculation; _S: spike viral inoculation.

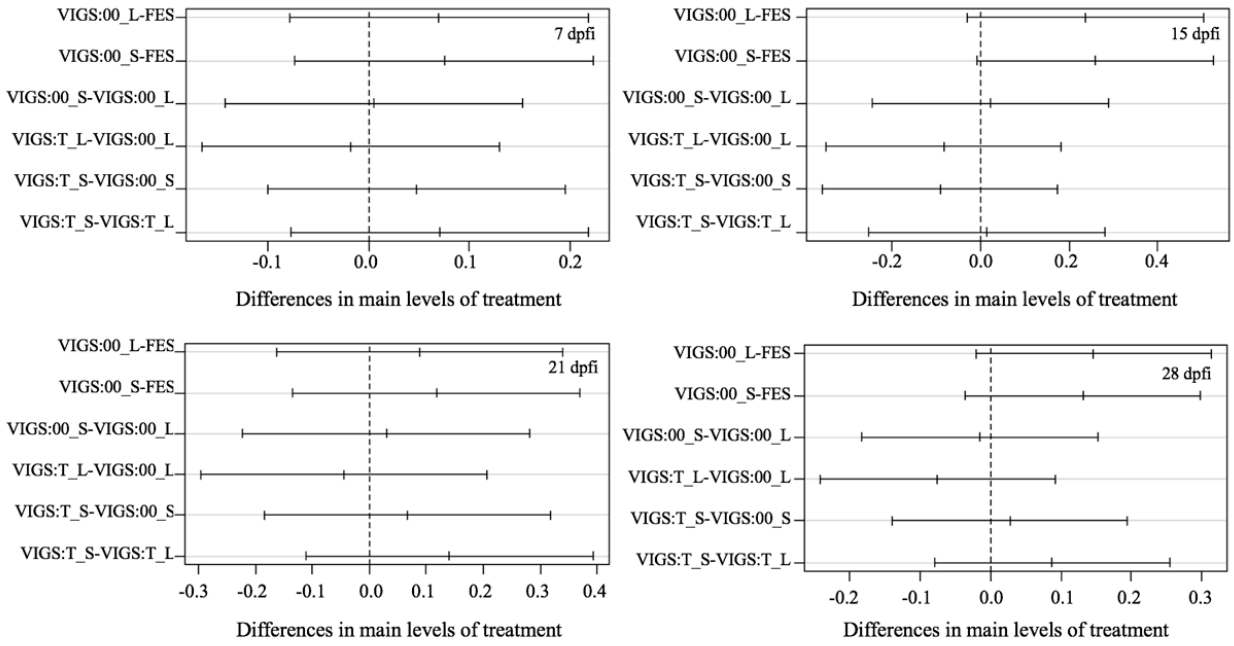

Supplementary Figure S11. Graphics showing the results of Turkey multiple comparisons of means between VIGS treatments at 95% family-wise confidence level for Y1193-06 in 7, 15, 21 and 28 days post *Fusarium* inoculation (dpfi).

Supplementary Table S1: Measurement and statistical analysis of fluorescence strength from the Western Blots of WFhb1-1 protein isolated from *Fusarium graminearum*- or water-inoculated spikelets of *Qfhb1*-nesr-isogenic line pair NIL-R (carrying *Qfhb1*) and NIL-S (not carrying *Qfhb1*) detected with anti-WFhb1-1 antibody PA-2. (hpfi: hours post *Fusarium* inoculation)

NIL-R	Water-inoculated						*Fusarium graminearum*-inoculated					
	0 hpfi	8 hpfi	12 hpfi	24 hpfi	36 hpfi	48 hpfi	0 hpfi	8 hpfi	12 hpfi	24 hpfi	36 hpfi	48 hpfi
Rep 1	0.292	0.375	0.505	0.541	0.517	0.529	0.292	0.753	0.637	0.831	0.749	0.941
Rep 2	0.359	0.437	0.436	0.414	0.587	0.807	0.359	0.37	0.373	0.651	0.447	0.731
Rep 3	0.457	0.414	0.598	0.572	0.515	0.580	0.457	0.504	0.335	0.203	0.692	0.926
Rep 4			0.235						0.285			
Rep 5			0.444						0.261			
Average	0.369	0.409	0.444	0.509	0.540	0.660	0.369	0.542	0.378	0.562	0.629	0.866
SD	0.083	0.031	0.133	0.084	0.041	0.184	0.083	0.194	0.151	0.323	0.160	0.117
SE	0.048	0.018	0.060	0.048	0.024	0.106	0.048	0.112	0.068	0.187	0.093	0.068
T-test	0.171							0.305	0.489	0.789	0.401	0.177
Normalized	0.369											
Fold change	1.000	1.016	1.369	1.466	1.401	1.434	1.000	2.041	1.726	2.252	2.030	2.550
		1.184	1.182	1.122	1.591	2.358		1.003	1.011	1.764	1.211	1.981
		1.122	1.621	1.550	1.396	1.572		1.366	-1.101	-1.818	1.875	2.509
			-1.570						-1.295			
			1.203						-1.414			
Ave	0.000	1.107	0.761	1.379	1.463	1.788	0.000	1.470	-0.215	0.733	1.706	2.347
SD	0.000	0.085	1.315	0.227	0.111	0.498	0.000	0.527	1.471	2.222	0.435	0.317
SE	0.000	0.049	0.588	0.131	0.064	0.288	0.000	0.304	0.658	1.283	0.251	0.183

NIL-S	Water-inoculated						*Fusarium graminearum*-inoculated					
	0 hpfi	8 hpfi	12 hpfi	24 hpfi	36 hpfi	48 hpfi	0 hpfi	8 hpfi	12 hpfi	24 hpfi	36 hpfi	48 hpfi
Rep 1	0.384	0.135	0.157	0.368	0.275	0.357	0.384	0.229	0.107	0.204	0.232	0.200
Rep 2	0.521	0.324	0.341	0.109	0.479	0.432	0.521	0.360	0.197	0.231	0.217	0.617
Rep 3	0.609	0.459	0.388	0.477	0.438	0.548	0.609	0.441	0.209	0.418	0.557	0.510
Rep 4			0.434						0.168			
Rep 5			0.402						0.123			
Average	0.505	0.306	0.344	0.318	0.397	0.446	0.505	0.343	0.161	0.284	0.335	0.442
SD	0.113	0.163	0.110	0.189	0.108	0.096	0.113	0.107	0.045	0.117	0.192	0.217
SE	0.065	0.094	0.049	0.109	0.062	0.056	0.065	0.062	0.020	0.067	0.111	0.125
T-test	0.171							0.757	0.009	0.235	0.112	0.982
Normalized	0.505											
Fold change	1.000	-3.741	-3.217	-1.371	-1.835	-1.414	1.000	-2.205	-4.720	-2.475	-2.177	-2.525
		-1.559	-1.481	-1.481	-4.630	-1.053		-1.403	-2.563	-2.186	-2.327	1.222
		-1.100	-1.302	-1.058	-1.152	1.086		-1.145	-2.416	-1.208	1.103	1.010
			-1.164						-3.006			
			-1.256						-4.106			
Ave	0.000	-2.133	-1.684	-2.353	-1.347	-0.499	0.000	-1.584	-3.362	-1.957	-1.134	-0.098
SD	0.000	1.411	0.865	1.978	0.426	1.378	0.000	0.553	1.007	0.664	1.938	2.105
SE	0.000	0.815	0.387	1.142	0.246	0.796	0.000	0.319	0.450	0.383	1.119	1.215

Supplementary Table S2. Measurements and statistical analysis of deoxynivalenol in the kernels harvested from *Fusarium*-inoculated spikes of *Qfhhb1* near-isogenic lines NIL-R (carrying *Qfhb1*) and NIL-S (not-carrying *Qfhb1*) inoculated with *WFhb1-1*-overexpressing BSMV:W, wildtype BSMV:00 or viral inoculation buffer FES.

NIL-S	BSMV:W	BSMV:00	FES	NIL-R	BSMV:W	BSMV:00	FES
**Exp1**	1.215774	14.07535	6.161836	**Exp1**	0.655526	0.300615	0.934874
	3.126315	41.69496	2.220297		2.065056	0.190049	0.350029
	11.98365	11.29983	8.33749		0	0.199467	1.37028
	5.028774	8.09783	13.21569		0.31215	0.096184	0.087819
	7.571769	0.587178	7.344306		3.774041	16.96128	6.746974
	2.939713	9.612612	1.192799		0	3.596712	2.419767
	5.81554	32.33572	6.273164		0	0.070522	0.056148
	0.421495	14.13058	8.351239		0.079412	1.968261	0.053421
	6.26241	1.524113	16.37352		17.64183	7.279542	0.53508
	11.91572	10.81543	18.52021		0	0	0.484565
	15.83737	4.594647	3.13002		8.458574	1.65449	2.723375
	0.327753	40.37845	2.988545		0.072951	2.848856	2.110902
	0	13.50766	15.24438		0	0	0
	5.520804	8.706757	4.964507		0	0	0
	5.605407	46.2993	7.833133		0	6.770157	0
	0.426047	6.076089	4.562935				
	0	0.741639	6.244772				
	0.41127	0	12.87445				
	0.392817	0.586586	10.42028				
	2.249001	8.936259	1.290686				
**Exp2**	0.809095	7.898423	14.65127	**Exp2**	2.435716	1.362592	0.170926
	1.742926	6.74183	27.95262		0	3.882156	0.772399
	3.155238	4.035355	2.723276		0	7.720536	0.229194
	3.055736	2.311778	32.81303		0.605462	1.359589	0
	1.239757	13.06527	0.320438		0.211784	0	1.946175
	0	0.739216	31.27817		0.248218	1.64968	0.86921
	0	10.08995	6.424545		0.893988	0.143852	0.790312
	0	0	0		1.662322	0.25483	8.70344
	0	0.746012	0		0	12.57758	2.455242
	0	0	0		0.025349	2.783813	1.121645
**Ave**	3.235146	10.65429	9.123587		1.565695	2.94683	1.397271
**SD**	4.042492	12.59088	8.828327		3.821606	4.303079	2.108828
**SE**	0.738055	2.29877	1.611825		0.764321	0.860616	0.421766
**T-test:**							
**Exp1**		0.009181	0.09878			0.734362	0.219431
**Exp2**		0.031289	0.146591			0.0645	0.345112
**Combined**		0.003741	0.593976			0.236058	0.112474

Supplementary Table S4. Formulas for reagents and the culture media used in this study.

Low salt LB (Luria-Bertani) Medium:
1% Tryptone, 0.5% Yeast Extract, 0.5% NaCl, pH 7.5 adjusted with 1 N NaOH.
For LB Agar plates: 15 g/liter agar added before autoclaving.

YPD or YEPD (Yeast Extract Peptone Dextrose Medium):
1% yeast extract, 2% peptone, 2% dextrose (glucose)

10X YNB (Yeast Nitrogen Base):
13.4% Yeast Nitrogen Base with Ammonium Sulfate without amino acids

500X B (0.02% Biotin):
Dissolve 20 mg biotin in 100 ml of water and filter sterilize. Store at 4°C.The shelf life of this solution is approximately one year.

10X M (5% Methanol):
Mix 5 ml of methanol with 95 ml of water. Filter sterilize and store at 4°C. The shelf life of this solution is approximately two months.

MGY (Minimal Glycerol Medium):
1.34% YNB, 1% glycerol, 4×10^{-5}% biotin
Combine aseptically 800 ml autoclaved water with 100 ml of 10X YNB, 2 ml of 500X B, and 100 ml of 10X GY. Store at 4° C. The self-life of this solution is approximately two months.

MM (Minimum Methanol):
1.34% YNB, 4 × 10–5% biotin, 0.5% methanol.
For liquid medium, autoclave 800 ml of water for 20 minutes on liquid cycle. Cool autoclaved water to 60°C and add: 100 ml of 10X YNB, 2 ml of 500X B, 100 ml of 10X M.
MM stores well for several months at 4°C.

Breaking buffer:
50 mM sodium phosphate (pH 7.4), 1 mM protease inhibitors, 1 mM EDTA, 5% glycerol.

2X Laemmli buffer:
0.125 M Tris-HCl (pH 6.8), 20% glycerol, 4% SDS, 0.004% Bromophenol blue, 10% beta-mercaptoethanol.

TBST (Tris-buffered Saline with Tween 20) buffer:
20 mM Tris (pH 7.5), 150 mM Nacl, 0.1% Tween 20

Blocking buffer:
5% dry milk powder in TBST buffer

FES buffer:
Sodium-pyrophosphate [1%, wt/vol], macaloid [1%, wt/vol], celite [1%, wt/vol], 0.5 M glycine, and 0.3 M K2HPO4, pH 8.5, with phosphoric acid.
